# A Systematic Review on Antimicrobial Resistance among *Salmonella* Typhi Worldwide

**DOI:** 10.4269/ajtmh.20-0258

**Published:** 2020-09-28

**Authors:** Christian S. Marchello, Samuel D. Carr, John A. Crump

**Affiliations:** Centre for International Health, University of Otago, Dunedin, New Zealand

## Abstract

Understanding patterns and trends of antimicrobial resistance (AMR) in *Salmonella* Typhi can guide empiric treatment recommendations and contribute to country decisions about typhoid conjugate vaccine (TCV) introduction. We systematically reviewed PubMed and Web of Science for articles reporting the proportion of *Salmonella* Typhi isolates resistant to individual antimicrobials worldwide from any time period. Isolates resistant to chloramphenicol, ampicillin, and trimethoprim–sulfamethoxazole were classified as multidrug resistant (MDR), and isolates that were MDR plus resistant to a fluoroquinolone and a third-generation cephalosporin were extensively drug resistant (XDR). Among the 198 articles eligible for analysis, a total of 55,459 *Salmonella* Typhi isolates were tested for AMR (median 80; range 2–5,191 per study). Of isolates from 2015 through 2018 in Asia, 1,638 (32.6%) of 5,032 were MDR, 167 (5.7%) of 2,914 were resistant to third-generation cephalosporins, and 148 (8.3%) of 1,777 were resistant to azithromycin. Two studies from Pakistan reported 14 (2.6%) of 546 isolates were XDR. In Africa, the median proportion of *Salmonella* Typhi isolates that were MDR increased each consecutive decade from 1990 to 1999 through 2010 to 2018. *Salmonella* Typhi has developed resistance to an increasing number of antimicrobial classes in Asia, where XDR *Salmonella* Typhi is now a major threat, whereas MDR has expanded in Africa. We suggest continued and increased surveillance is warranted to inform empiric treatment decisions and that AMR data be incorporated into country decisions on TCV introduction.

## INTRODUCTION

Typhoid fever is a systemic infection caused by the bacterium *Salmonella enterica* subspecies *enterica* serovar Typhi (*Salmonella* Typhi) and is an important cause of illness and death worldwide, with an estimated 10.9 million new infections and 116,800 typhoid fever–related deaths occurring annually.^1–3^ Typhoid fever is difficult to distinguish from other causes of febrile illnesses, compounded by limited laboratory services in some low- and middle-income countries, making specific diagnosis and appropriate antimicrobial treatment challenging in routine practice.^4–6^

Historically, chloramphenicol, ampicillin, and trimethoprim–sulfamethoxazole were the first-line antimicrobial treatments for typhoid fever.^7^ However, multidrug resistant (MDR) *Salmonella* Typhi, defined as resistance to these three first-line drugs,^8^ was widespread by the late 1980s and 1990s, with reports from Pakistan, India, and other southern and Southeast Asian countries.^9^ Subsequently, ciprofloxacin became the drug of choice, but resistance appeared quickly, first in 1991^10^ and in an outbreak in 1997.^11^ With the emergence of MDR *Salmonella* Typhi and MDR with fluoroquinolone resistance, third-generation cephalosporins, macrolides, and carbapenems have been used increasingly for the treatment of typhoid fever.^7^ Extensively drug resistant (XDR) *Salmonella* Typhi, defined as resistance to first-line antimicrobials, a fluoroquinolone, and a third-generation cephalosporin,^12^ was reported in Hyderabad, Pakistan, in 2016.^13^ Since then, the WHO has been notified of more than 10,365 infections with XDR *Salmonella* Typhi in Pakistan,^14^ and travel-associated infections have been reported in Canada,^15^ Denmark,^16^ Australia,^17^ and the United States.^18^ Hence, antimicrobial resistance (AMR) in *Salmonella* Typhi is a global threat.

Increasing access to safe drinking water, food, and improved sanitation are important measures for controlling the impact and spread of typhoid fever,^19–21^ but have elsewhere been associated with socioeconomic progress that has taken place over long time periods.^22–24^ Typhoid vaccines represent an important and accessible tool to avert illness and death in the short- to medium-term while water, food, and sanitation improvements take place. To assist countries with decision-making about typhoid conjugate vaccine (TCV) introduction and other control efforts, and to guide empiric management decisions for typhoid fever, we performed a comprehensive, systematic review of the literature to describe the prevalence and trends of AMR among *Salmonella* Typhi.

## METHODS

### Search strategy and study selection.

The systematic review protocol was registered with PROPSERO: International Prospective Register of Systematic Reviews (CRD42019131038) on May 10, 2019. Following the preferred reporting items for systematic reviews and meta-analyses,^25^ we searched two databases, PubMed from inception through April 16, 2019, and Web of Science from inception through April 17, 2019. Each search included key words of Typhi, typhoid, “enteric fever,” antimicrobial, susceptibility, and resistance (Supplemental Table S1). No restrictions were placed on location, date of specimen collection, or language of publication.

Epidemiologic studies of any design reporting antimicrobial susceptibility testing (AST) results of *Salmonella* Typhi isolated from human source, normally sterile site (e.g., blood, bone marrow) specimens, were included. Policy reports, commentaries, editorials, and conference abstracts were excluded, as were studies where we could not distinguish between isolates from normally sterile site specimens and isolates not from normally sterile site specimens (e.g., stool, urine). Studies that did not present sufficient detail to calculate the prevalence of AMR among all reported *Salmonella* Typhi isolates were also excluded. Studies reporting travel-associated infections were included only if we were able to identify the country where the infection was likely acquired.

Search results from each database were imported into Endnote X8 (Clarivate Analytics, Boston, MA). We also included studies identified from the reference lists of three previous reviews on bloodstream infections that also reported AST of *Salmonella* Typhi.^26–28^ Endnote was used to remove duplicates before a de-duplicated list of articles was uploaded to the online systematic review tool Rayyan (Qatar Computing Research Institute, Doha, Qatar).^29^ All subsequent processes were performed in parallel by two authors (C. S. M. and S. D. C.). We screened titles and abstracts for inclusion, and any article selected by at least one author was included for full-text review. We then screened each full-text article for inclusion, with discrepancies resolved through discussion or the involvement of a third author (J. A. C.). After establishing a final list of included full-text articles, two authors abstracted study characteristics and AST data in parallel using a shared Google Sheets spreadsheet (Google LLC, Mountain View, CA).

### Data abstraction.

Study characteristics that were abstracted included first author, publication year, normally sterile specimen type, and country of specimen collection. When a study collected isolates from multiple locations within a country or in separate countries, we documented the additional locations as study sites. We abstracted data for the year of susceptibility testing; antimicrobial susceptibility testing (AST) interpretive criteria used, such as the Clinical and Laboratory Standards Institute (CLSI) or European Committee on AST, and year of criteria; AST method (e.g., agar dilution, disk diffusion); number of total isolates tested; and number of isolates that tested susceptible, intermediate, or resistant to a predefined list of antimicrobials. The predefined list was compiled from the CLSI M100 table of suggested antimicrobial agents for Enterobacteriaceae groups A and B.^30^ Because zone sizes or minimum inhibitory concentration (MIC) values were often not reported, we were unable to attempt to recategorize AST results for fluoroquinolones, third-generation cephalosporins, and azalides for the current CLSI interpretive criteria.^31^

In addition to recording susceptibility results to individual antimicrobials, data were also abstracted on the MDR phenotype, defined as resistance to chloramphenicol, ampicillin, and trimethoprim–sulfamethoxazole^9^; and the XDR phenotype, defined as resistance to ampicillin, chloramphenicol, trimethoprim–sulfamethoxazole, a fluoroquinolone, and a third-generation cephalosporin.^12^ Fluoroquinolones abstracted were ciprofloxacin or ofloxacin, and third-generation cephalosporins were ceftriaxone or cefotaxime. We subsequently classified study sites by UN geographic regions and subregions.^32^

### Analyses.

For each antimicrobial tested, we divided the number of resistant isolates by the total number of isolates tested and multiplied the resulting fraction by 100 to produce the proportion (%) of resistant isolates. When only the number of susceptible isolates was supplied and resistant isolates were not explicitly provided, we assumed that non-susceptible isolates were resistant. For example, an article described 90.0% of 100 isolates were susceptible to ampicillin, and we imputed data that 10 isolates in that study were resistant. No other data were imputed. Median proportions of isolates resistant by UN region and the chi-squared test for trend in proportions over four time periods (1970–1989, 1990–1999, 2000–2009, and 2010–2018) were calculated in R version 3.6.1 (R Foundation for Statistical Computing, Vienna, Austria). Time periods were selected by decade, combining the 1970s and 1980s because of limited data available during those years. Time series histograms using 5-year interval periods were produced in Microsoft Excel 2016 (Microsoft Corporation, Redmond, WA) to illustrate the emergence of resistance patterns over time globally, in Asia, and in Africa. Intervals of 5 years were chosen for histograms to produce a more detailed time series than every 10 years. Maps were created using an online open-source map tool.^33,34^ In keeping with the aim to aid country-based decisions on TCV introduction and guide empiric management of typhoid fever, the decision to not perform a meta-analysis was made to avoid pooling data of multiple drugs from multiple countries. As a secondary analysis of published data, this study was exempt from institutional review board approval.

## RESULTS

**Figure 1.**
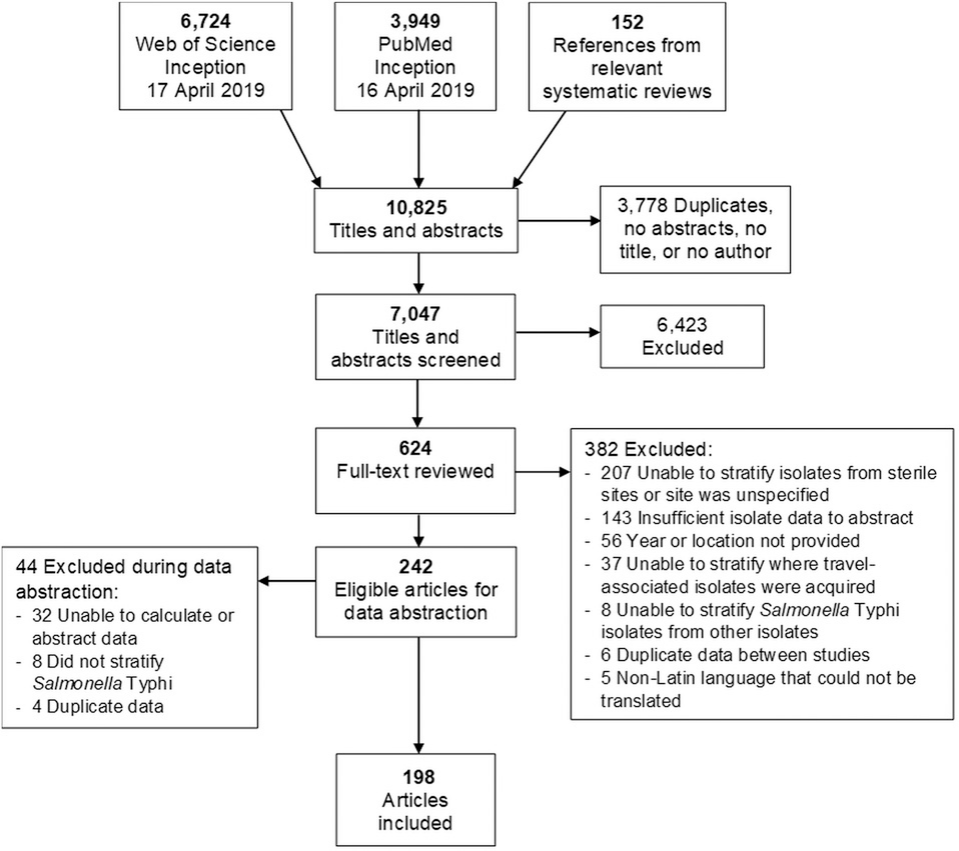
Preferred reporting items for systematic reviews and meta-analyses flow diagram of search strategy and selection of articles for antimicrobial resistance in *Salmonella* Typhi, 1972–2018. Some articles met the multiple exclusion criteria, and thus the sum of exclusion reasons is greater than the number of articles excluded.

Our search of PubMed and Web of Science returned 6,724 and 3,949 articles, respectively (Figure 1). When including the references of relevant systematic reviews and then removing duplicates, we screened 7,047 titles and abstracts. Of these, 624 (8.9%) full-text articles were eligible for further review. We excluded 426 articles. The most common reason for article exclusion was failure to distinguish isolates that were collected from normally sterile sites from other sites, or the site was unspecified, resulting in 198 articles eligible for analysis (Supplemental Table S2).

### Study characteristics.

**Figure 2.**
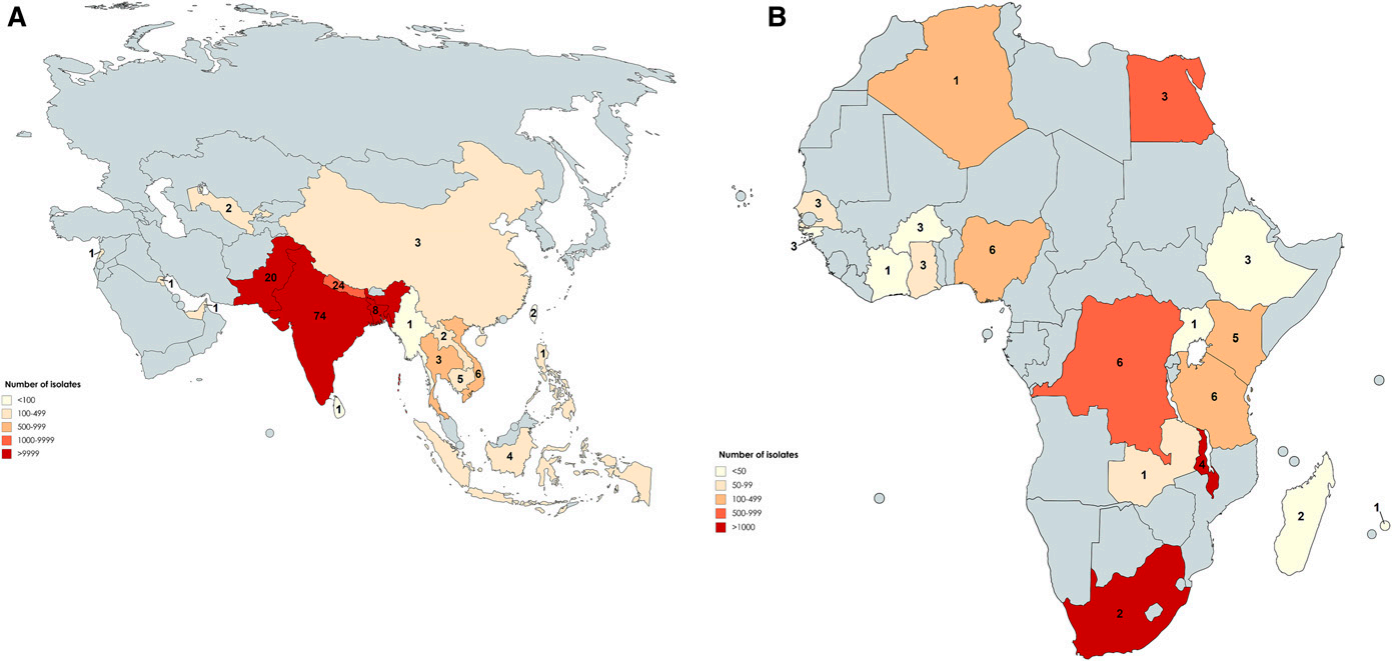
(**A**) Number of study sites and isolates tested by country in the Asia region, 1972–2018 (created with MapChart). Number for each country denotes the number of study sites that reported data for *Salmonella* Typhi resistance. (**B**) Number of study sites and isolates tested by country in the Africa region, 1972–2018 (created with MapChart). Number for each country denotes the number of study sites that reported data for *Salmonella* Typhi resistance.

Studies reported on isolates collected from 1972 through 2018 in 38 countries and tested a total of 55,459 *Salmonella* Typhi isolates for AMR. By study, a median (range) of 80 (2–5,191) *Salmonella* Typhi isolates were tested. Among the 198 studies, four (2.0%) reported data collection in multiple locations, resulting in 216 study sites; 159 (73.6%) of 216 were located in 18 countries in the Asia region (Figure 2A), 54 (25.0%) in 18 countries in Africa (Figure 2B), two (0.9%) in the Americas, and one (0.5%) in Europe. There were 127 (58.8%) sites located in Southern Asia: 74 (34.3%) in India, 24 (11.1%) in Nepal, and 20 (9.3%) in Pakistan. Among isolates tested, 47,145 (85.0%) of 55,459 were from study sites located in Asia, 8,249 (14.9%) were from Africa, 43 (0.1%) were from the Americas, and 22 (< 0.1%) from Europe. Among isolates from Asia, 43,870 (93.1%) of 47,145 were from the Southern Asia subregion.

Of the 198 studies, 130 (65.7%) reported AST using CLSI interpretive criteria (Table 1). Among studies using CLSI criteria, 82 (63.1%) were before the 2012 breakpoint changes for fluoroquinolones.^35^ Ten (5.1%) studies used a combination of two AST interpretive criteria, and 59 (29.8%) studies did not report which AST interpretive criteria were used. Regarding laboratory antimicrobial susceptibility methods and findings, 173 (87.4%) of 198 studies used a disc diffusion method (e.g., the Kirby–Bauer test, Stokes’ method), whereas 82 (41.1%) used outputs from MIC techniques (e.g., broth micro-dilution, E-test, agar dilution) to determine resistant, intermediate, and susceptible isolates. Twelve (6.1%) used automated methods; 78 (39.4%) reported using a combination of a disc diffusion, MIC, or automated method; and nine (4.5%) did not report how susceptibility was determined.

**Table 1:** Interpretive criteria and laboratory testing method used among 198 included studies of antimicrobial resistance of *Salmonella* Typhi, global, 1972 through 2018

Interpretive criteria	Number of studies (% of 198 included*)
CLSI	83 (41.9)
Pre-2012	35 (17.7)
2012 or later	38 (19.2)
Specific year unreferenced†	10 (5.1)
Not reported	59 (29.8)
The National Committee for Clinical Laboratory Standards (now CLSI as of 2005)	47 (23.7)
The European Committee on Antimicrobial Susceptibility Testing	10 (5.1)
The British Society for Antimicrobial Chemotherapy	7 (3.5)
The French Microbiology Society	2 (1.0)
Laboratory antimicrobial susceptibility testing method	
Disc diffusion‡	173 (87.4)
Minimum inhibitory concentration§	82 (41.4)
Combination of at least two methods	78 (39.4)
Automated	12 (6.1)
Not reported	9 (4.5)

CLSI = Clinical and Laboratory Standards Institute.

* Some studies used multiple interpretive criteria or testing strategies for different antimicrobials; thus, numbers will exceed the total number of studies included.

† Two studies specified multiple CLSI years covering both pre-2012 and 2012 or later.

‡ The Kirby–Bauer test and the Stokes method.

§ E-test, agar dilution, and broth dilution.

### Overall AMR.

Among all *Salmonella* Typhi isolates, 9,056 (25.9%) of 34,996 were resistant to chloramphenicol, 13,481 (38.8%) of 34,783 to ampicillin, and 13,366 (37.9%) of 35,270 to trimethoprim–sulfamethoxazole (Table 2). Of isolates, 12,666 (35.5%) of 35,659 were MDR, 9,495 (64.7%) of 14,671 were nalidixic acid resistant, and 5,406 (15.0%) and 6,979 (19.4%) of 35,975 were ciprofloxacin resistant and intermediate, respectively. Of isolates, 450 (1.3%) of 35,302 were resistant to ceftriaxone and 270 (4.5%) of 6,043 to azithromycin.

**Table 2:** *Salmonella* Typhi antimicrobial susceptibility testing profiles, global, 1972–2018

Antimicrobial class and agent*	Susceptible	Intermediate	Resistant	Total tested	Percent of isolates resistant
Traditional first-line					
Chloramphenicol	25,907	33	9,056	34,996	25.9
Ampicillin	21,197	105	13,481	34,783	38.8
Amoxicillin	1,569	34	2,525	4,128	61.2
Amoxicillin–clavulanic acid	1,184	1	103	1,288	8.0
Trimethoprim–sulfamethoxazole	21,896	8	13,366	35,270	37.9
Quinolone					
Nalidixic acid	5,084	92	9,495	14,671	64.7
Fluoroquinolone					
Ciprofloxacin	23,590	6,979	5,406	35,975	15.0
Ofloxacin	8,095	389	4,106	12,590	32.6
Third-generation cephalosporin					
Ceftriaxone	34,771	81	450	35,302	1.3
Cefotaxime	5,072	45	468	5,585	8.4
Macrolide					
Azithromycin	5,759	14	270	6,043	4.5
Carbapenem					
Meropenem	813	0	21	834	2.5
Aminoglycoside					
Gentamicin†	5,477	16	676	6,169	11.0
Tetracycline	2,068	24	1,435	3,527	40.7
Multidrug resistant (MDR)	–	–	12,666	35,659	35.5
Extensively drug resistant (XDR)	–	–	14	546	2.6

MDR = phenotype defined as resistance to chloramphenicol, ampicillin, and trimethoprim–sulfamethoxazole. XDR = phenotype defined as resistance to ampicillin, chloramphenicol, trimethoprim–sulfamethoxazole, a fluoroquinolone, and a third-generation cephalosporin.

* Antimicrobial susceptibility profiles not recategorized to current Clinical and Laboratory Standards Institute break points.

† Susceptibility of gentamicin not recommended to be reported or used therapeutically for *Salmonella* Typhi by the Clinical Laboratory Standards Institute.

**Figure 3.**
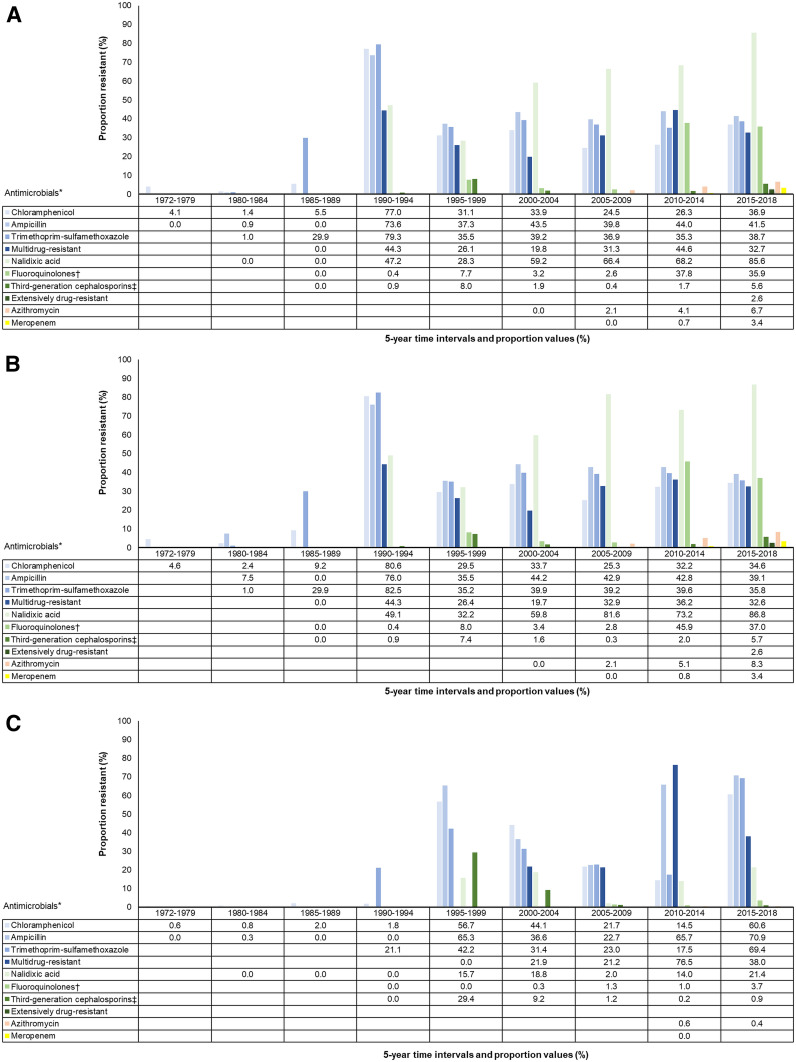
(**A**) Antimicrobial resistant *Salmonella* Typhi isolates worldwide, 1972–2018. (**B**) Antimicrobial resistant *Salmonella* Typhi isolates in Asia, 1972–2018. (**C**) Antimicrobial resistant *Salmonella* Typhi isolates in Africa, 1972–2018. Full data provided in Supplement Table S4. *Ordered chronologically by antimicrobial agent introduction and grouped by color by multidrug resistant and extensively drug resistant phenotypes. †Fluoroquinolone includes ciprofloxacin and ofloxacin. ‡Third-generation cephalosporin includes ceftriaxone and cefotaxime.

From 1990 through 1994, 955 (77.0%) of 1,241, 793 (73.6%) of 1,077, and 871 (79.3%) of 1,098 *Salmonella* Typhi isolates were resistant to chloramphenicol, ampicillin, and trimethoprim–sulfamethoxazole, respectively (Figure 3A). During the same period from 1990 through 1994, 1,205 (44.3%) of 2,719 were MDR. Of isolates from 2010 through 2014, 5,981 (44.6%) of 13,416 were MDR, and from 2015 through 2018 1,679 (32.7%) of 5,140 were MDR. Of isolates in the time periods 2005–2009, 2010–2014, and 2015–2018, 27 (2.1%) of 1,279, 93 (4.1%) of 2,263, and 150 (6.7%) of 2,247 were resistant to azithromycin, respectively.

### Antimicrobial resistance in Asia.

When stratified by time periods 1972–1989, 1990–1999, 2000–2009, and 2010–2018 in the Asia region, the median (interquartile range) proportion of *Salmonella* Typhi isolates that were MDR was 0.0% (0.0%), 31.2% (22.9–47.0%), 16.2% (6.1–35.6%), and 5.5% (2.0–24.3%), respectively (Table 3). By time period, ciprofloxacin resistance was found in zero (0.0%) of 61, 219 (3.7%) of 5,912, 439 (3.1%) of 14,040, and 4,670 (41.1%) of 11,349 (χ^2^ 5,376, *P* < 0.001) isolates. By time period, ceftriaxone resistance was found in zero (0.0%) of 203, 124 (2.5%) of 4,898, 47 (0.4%) of 12,761, and 266 (1.9%) of 13,970 (χ^2^ 2.3, *P* = 0.133) isolates.

**Table 3:** Proportion of *Salmonella* Typhi isolates resistant to antimicrobials in Asia over four time periods from 1972 through 2018

Antimicrobial*	1972–1989			1990–1999			2000–2009			2010–2018			
	*n*/*N*	%	Median proportion, % (IQR)	*n*/*N*	%	Median proportion, % (IQR)	*n*/*N*	%	Median proportion, % (IQR)	*n*/*N*	%	Median proportion, % (IQR)	χ^2^ Trend of proportion (*P*-value)
Chloramphenicol	210/4,525	4.6	4.0 (0.8–31.4)	1,530/3,134	48.8	57.6 (37.7–78.0)	2,418/8,149	29.7	20.8 (6.2–40.9)	3,770/11,261	33.5	5.7 (2.1–31.3)	649.9 (< 0.001)
Ampicillin	16/225	7.1	0.0 (0.0–5.1)	1,476/2,969	49.7	57.8 (36.4–68.8)	4,044/9,319	43.4	38.5 (17.0–53.6)	6,755/16,304	41.4	15.6 (5.0–47.7)	17.5 (< 0.001)
Trimethoprim–sulfamethoxazole	48/350	13.7	0.6 (0.0–9.0)	1,533/2,956	51.9	54.3 (33.5–80.9)	4,142/10,505	39.4	30.6 (9.9–50.0)	6,428/16,799	38.3	6.8 (3.0–34.9)	47.1 (< 0.001)
Multidrug resistant (MDR)	0/61	0.0	0.0 (0.0)	2,000/5,731	34.9	31.2 (22.9–47.0)	2,780/10,214	27.2	16.2 (6.1–35.6)	5,487/15,661	35.0	5.5 (2.0–24.3)	23.1 (< 0.001)
Nalidixic acid	NR	–	NR	390/874	44.6	24.5 (5.7–48.6)	5,338/7,360	72.5	75.0 (60.4–86.9)	3,667/4,868	75.3	93.1 (78.1–96.9)	185.9 (< 0.001)
Ciprofloxacin	0/61	0.0	0.0 (0.0)	219/5,912	3.7	0.0 (0.0–5.0)	439/14,040	3.1	0.0 (0.0–4.2)	4,670/11,349	41.1	19.5 (3.5–75.4)	5,376 (< 0.001)
Ceftriaxone	0/203	0.0	0.0 (0.0)	124/4,898	2.5	0.0 (0.0)	47/12,761	0.4	0.0 (0.0)	266/13,970	1.9	0.0 (0.0–0.1)	2.3 (0.133)
Azithromycin	NR	–	NR	NR	–	NR	27/1,533	1.8	0.0 (0.0)	238/3,556	6.7	0.0 (0.0–5.0)	51.8 (< 0.001)

IQR = interquartile range; MDR = phenotype defined as resistance to chloramphenicol, ampicillin, and trimethoprim–sulfamethoxazole; NR = no eligible studies reporting data for year period and drug.

* Antimicrobial susceptibility profiles not recategorized to current Clinical and Laboratory Standards Institute break points.

Of isolates from 2015 through 2018, 1,638 (32.6%) of 5,032 were MDR, 167 (5.7%) of 2,914 were resistant to third-generation cephalosporins, 148 (8.3%) of 1,777 to azithromycin, and 20 (3.4%) of 594 to meropenem (Figure 3B). Two studies from Pakistan reported 14 (2.6%) of 546 isolates were XDR.^36,37^

### Antimicrobial resistance in Africa.

Two studies tested 3,327 isolates during the time period 1972 through 1989, and neither study reported testing trimethoprim–sulfamethoxazole, MDR, ciprofloxacin, ceftriaxone, or azithromycin (Table 4).^38,39^ In the time periods 1990–1999, 2000–2009, and 2010–2018, the median (interquartile range) proportion of MDR *Salmonella* Typhi isolates was 0.0% (0.0%), 0.0% (0.0–25.5%), and 38.0% (30.4–85.7%), respectively. Of isolates during the same three time periods, ciprofloxacin resistance was found in zero (0.0%) of 133, 14 (1.2%) of 1,209, and 39 (1.2%) of 3,228 (χ^2^ 0.7, *P* = 0.415), respectively. Of isolates, ceftriaxone resistance was found in zero (0.0%) of 55, eight (0.7%) of 1,160, and five (0.2%) of 2,212 (χ^2^ 2.9, *P* = 0.09), respectively. No eligible studies in Africa reported azithromycin resistance until the 2010–2018 period. Of isolates in 2010–2018, five (0.5%) of 954 were azithromycin resistant. One study reported zero (0.0%) of seven isolates resistant to meropenem in 2013.^40^

**Table 4:** Proportion of *Salmonella* Typhi isolates resistant to antimicrobials in Africa over four time periods from 1972 through 2018

	1972–1989			1990–1999			2000–2009			2010–2018			χ^2^ Trend of proportion (*P*-value)
Antimicrobial*	*n*/*N*	%	Median proportion, % (IQR)	*n*/*N*	%	Median proportion, % (IQR)	*n*/*N*	%	Median proportion, % (IQR)	*n*/*N*	%	Median proportion, % (IQR)	
Chloramphenicol	6/3,313	0.2	0.0 (0.0–0.2)	81/157	51.6	0.0 (0.0–0.0)	319/1,276	25.0	49.7 (4.9–97.6)	784/1,155	67.9	66.7 (0.0–74.1)	2,251.9 (< 0.001)
Ampicillin	35/3,313	1.1	0.6 (0.4–1.7)	69/177	39.0	0.0 (0.0–2.1)	294/1,206	24.4	31.8 (5.8–72.5)	730/3,166	23.1	33.3 (0.0–76.7)	675.7 (< 0.001)
Trimethoprim–sulfamethoxazole	NR	–	NR	55/159	34.6	0.0 (0.0)	310/1,273	24.4	40.4 (7.7–80.0)	850/3,163	26.9	49.4 (0.2–79.9)	0.002 (0.996)
Multidrug resistant (MDR)	NR	–	NR	0/37	0.0	0.0 (0.0)	226/1,060	21.3	0.0 (0.0–25.5)	2,173/2,895	75.1	38.0 (30.4–85.7)	976.6 (< 0.001)
Nalidixic acid	0/14	0.0	0.0 (0.0)	11/95	11.6	0.0 (0.0–13.2)	29/1,059	2.7	1.5 (0.0–1)	51/358	14.2	15.4 (3.5–21.4)	25.5 (< 0.001)
Ciprofloxacin	NR	–	NR	0/133	0.0	0.0 (0.0)	14/1,209	1.2	0.0 (0.0)	39/3,228	1.2	0.0 (0.0–4.0)	0.7 (0.415)
Ceftriaxone	NR	–	NR	0/55	0.0	0.0 (0.0)	8/1,160	0.7	0.0 (0.0)	5/2,212	0.2	0.0 (0.0)	2.9 (0.09)
Azithromycin	NR	–	NR	NR		NR	NR		NR	5/954	0.5	0.0 (0.0–0.5)	†

IQR = interquartile range; NR = no eligible study reporting data for year period and drug.

* Antimicrobial susceptibility profiles not recategorized to current Clinical and Laboratory Standards Institute break points.

† Unable to calculate; MDR = phenotype defined as resistance to chloramphenicol, ampicillin, and trimethoprim–sulfamethoxazole.

Among studies reporting MDR isolates, one study reported zero (0.0%) of 37 MDR isolates from 1995 through 1999.^41^ Of isolates during the periods 2000–2004, 2005–2009, and 2010–2014, 28 (21.9%) of 128, 198 (21.2%) of 932, and 2,132 (76.5%) of 2,787 isolates were MDR, respectively (Figure 3C). From 2015 through 2018, one study in Democratic Republic of the Congo reported 41 (38.0%) of 108 isolates were MDR.^42^ Among isolates from 2015 through 2018, 357 (60.6%) of 589 were resistant to chloramphenicol, 343 (70.9%) of 484 to ampicillin, and 396 (69.4%) of 571 to trimethoprim–sulfamethoxazole. Third-generation cephalosporin resistance was found in 35 (29.4%) of 119, 24 (9.2%) of 262, 13 (1.2%) of 1,111, four (0.2%) of 2,416, and one (0.9%) of 111 in 1995–1999, 2000–2004, 2005–2009, 2010–2014, and 2015–2018, respectively. No eligible studies from Africa reported XDR isolates.

### Other typhoidal and non-typhoidal *Salmonella.*

Among 12,850 other typhoidal *Salmonella* isolates tested, 10,464 (81.4%) were *Salmonella* serovar Paratyphi A, 1,305 (10.2%) *Salmonella* Paratyphi A or Paratyphi B, 1,012 (7.9%) unspecified *Salmonella* “Paratyphi,” 68 (0.5%) *Salmonella* Paratyphi B, and one (< 0.1%) *Salmonella* Paratyphi C. Of 1,462 non-typhoidal *S. enterica* isolates tested, 793 (54.2%) were *Salmonella* Typhimurium and 669 (45.8%) were *Salmonella* Enteritidis. Of *Salmonella* Paratyphi A isolates, 650 (8.9%) of 7,335 were MDR, 3,027 (32.4%) of 9,332 were resistant to fluoroquinolones, and 45 (2.1%) of 2,184 were resistant to azithromycin. Antimicrobial susceptibility testing data for typhoidal *Salmonella* other than serovar Typhi are presented in Supplemental Table S4.

## DISCUSSION

We demonstrate that AMR among *Salmonella* Typhi isolates is a substantial problem in countries in Asia and Africa. Multidrug resistant *Salmonella* Typhi remains prevalent in Asia, with resistance developing to an increasing number of antimicrobial classes such that XDR *Salmonella* Typhi is now a major threat. MDR *Salmonella* Typhi is a growing problem in Africa.

The first AMR phenotype to appear in our review was resistance to chloramphenicol in a study from Vietnam in 1972.^43^ We show clear evidence of increasing prevalence of resistance to traditional first-line antimicrobials in the 1980s and a substantial increased prevalence of MDR in the 1990s. Because third-generation cephalosporins and azithromycin were not widely used or available for the treatment of typhoid fever from 1970 through 1989,^7,44^ data were lacking on susceptibility to these drugs during earlier time periods.

When stratified by region, resistance to the three traditional first-line antimicrobials and MDR appear later in Africa compared with Asia. This difference is consistent with phylogenetic analyses of whole-genome sequencing data, suggesting the introduction of the MDR H58 haplotype from Asia to Africa.^45–47^ As there were no eligible studies located in Oceania, we could not address AMR in this region. The H58 haplotype was identified in Fiji as early as 1992.^45^ However, the H58 haplotype has so far not expanded in Fiji where most *Salmonella* Typhi isolates remain susceptible to traditional first-line antimicrobials.^48,49^ We observed a decline in the median prevalence of resistance to each of the traditional first-line antimicrobials and in MDR across Asia, corroborating reports of increasing prevalence of chloramphenicol-susceptible strains in areas that previously documented a high prevalence of resistance to the drug.^50–53^

The trend of resistance to a growing number of antimicrobial classes is alarming, including recently to azithromycin^37,54,55^ and outbreaks of XDR *Salmonella* Typhi.^12–14^ Our review captured one study in Pakistan^36^ and one in Indonesia^56^ reporting meropenem resistance in *Salmonella* Typhi. To our knowledge, these would be the first reports of carbapenem resistance in *Salmonella* Typhi and cause for great concern. We recommend that this finding be confirmed with additional testing of the isolates. There were no eligible articles in our review reporting XDR *Salmonella* Typhi from Africa. However, if the history of the spread of MDR *Salmonella* Typhi applies, XDR *Salmonella* Typhi is likely to be introduced and spread in Africa in due course. Resistance to multiple antimicrobial classes is likely being driven by indiscriminate antimicrobial use, and weak stewardship practices in the community and in healthcare facilities.^57^

Because we sought to produce a comprehensive review of AMR to assist countries with decision-making about TCV introduction and to guide empiric management decisions, we decided *a priori* against conducting a meta-analysis that would produce pooled prevalence estimates. Furthermore, because the overwhelming majority of eligible studies were located in Southern Asia and reported a broad range of antimicrobials, pooled estimate would be of little value when evaluating AMR in *Salmonella* Typhi for a specific country that was not part of the pooled analysis.

An earlier systematic review and meta-analysis of enteric fever produced pooled estimates for MDR and fluoroquinolone resistance.^58^ The earlier review and ours both demonstrate that although data are limited for known typhoid-endemic areas, antimicrobial resistant *Salmonella* Typhi is becoming more prevalent. However, our review also had a number of additional strengths. First, Browne and others classified intermediate organisms as resistant. According to the CLSI, the intermediate category “implies clinical efficacy in anatomical sites where the drugs are physiologically concentrated” and the resistant category “implies clinical efficacy of the agent against the isolate has not been reliably shown in treatment studies.”^59^ In keeping with this guidance, we did not categorize intermediate as a category of resistant isolates, and thus avoided possible bias toward inflation in the number of resistant isolates. Second, we classified isolates as MDR if the study authors clearly defined MDR isolates as resistant to chloramphenicol, ampicillin, and trimethoprim–sulfamethoxazole, and we could ascertain that resistance to these three first-line antimicrobials was present. Third, we placed no restrictions on the number of isolates the study tested for resistance. Fourth, we required studies to report the dates for data collection. If a study did not report dates, it was excluded, and we did not impute dates based on study publication date. By not pooling the proportions, recategorizing intermediate isolates, or imputing data, we believe our review presents raw data that are readily accessible to decision-makers.

Our study had a number of limitations. First, a substantial proportion of included studies did not report the interpretive criteria used, and we were unable to make adjustments to the interpretive criteria based on the current CLSI or other breakpoint guidelines because we did not have access to zone sizes or MIC values for most studies. Notably, the CLSI interpretive criteria for *Salmonella* Typhi have changed over time, including to fluoroquinolones, third-generation cephalosporins, and the introduction of azalide break points.^31^ These changes may have generated artefactual variation in the prevalence of resistance over time. Second, without having a full classification of susceptible, intermediate, and resistant isolates in every study, we were also limited by having to impute data on resistant isolates in studies that only reported those that were susceptible or that only provided the proportion and total number of isolates tested. Third, our data comprised mostly hospital and healthcare facility–based studies that may overrepresent AMR. Finally, our global AMR data are driven by the predominance of studies located in Asia, particularly India, Nepal, and Pakistan. Several UN regions and subregions had no eligible studies or were underrepresented. Higher income countries in Northern America and Northern, Southern, and Western Europe usually rely on national surveillance systems to report AMR.^60,61^ No eligible studies were identified from Oceania, Central America, Caribbean, or Eastern Europe. Although studies were included from South America, Northern, Middle, and Southern Africa, and Eastern, Central, and Western Asia, these were limited in number and dominated by studies in Eastern Africa, and Southeastern and Southern Asia.

The prevalence of resistance among *Salmonella* Typhi isolates is growing, especially the XDR phenotype in Asia and the MDR phenotype in Africa. Isolates resistant to many classes of antimicrobials pose a substantial threat to global health. Typhoid control efforts should be expanded, including the introduction of TCV which has been demonstrated to reduce typhoid fever incidence in endemic areas^62^ and was recommended to combat and control XDR.^63^ Where XDR *Salmonella* Typhi is present, azithromycin and carbapenems remain effective for uncomplicated and complicated typhoid fever, respectively. We encourage the implementation of robust stewardship and surveillance programs to inform empiric treatment decisions and reduce AMR.

## Supplemental tables

Supplemental materials
