# Supplementary material for: A Systematic Review on Antimicrobial Resistance among Salmonella Typhi Worldwide
Source: Am J Trop Med Hyg. 2020 Sep 28;103(6):2518–27. doi: 10.4269/ajtmh.20-0258 (PMC7695120; doi:10.4269/ajtmh.20-0258)
Supplement: Supplementary file 1 [file tpmd200258.SD1.pdf]

Supplemental Table S1. Search strategies used for the systematic review on global antimicrobial resistance in *Salmonella* Typhi

**PubMed on 16 April 2019**<br><br>((typhi OR typhoid OR "enteric fever") AND (antimicrob* OR sensitiv* OR suscept* OR resistan* OR empiric*)) AND hasabstract[text]
**Web of Science on 17 April 2019**<br><br>TOPIC: (typhi) or TOPIC: (typhoid) or TOPIC: (“enteric fever”)<br><br>AND<br><br>TOPIC: (antimicrob*) or TOPIC: (sensitive*) or TOPIC: (suscept*) or TOPIC: (resistan*) or TOPIC: (empiric*)

*An asterisk is used in each database to search multiple suffixes, iterations, and spellings of a term.

Supplemental Table S2. Characteristics of 198 included articles of antimicrobial resistance in *Salmonella* Typhi, global, 1972-2018

United Nations region and sub-region	Country	Year(s) of data collection	Sterile sample type	Total *Salmonella* Typhi isolates	First author
**Africa**					
Eastern Africa	Kenya	1988-1999	blood	38	Kariuki [1]
	Kenya	1994	blood	24	Dougle [2]
	Malawi	1996-1997	blood	15	Walsh [3]
	Malawi	1997-1998	blood	12	Gordon [4]
	Mauritius (local); India, Pakistan, and Sri Lanka (travel)	1997-2004	blood	17	Issack [5]
	Malawi	1998	blood	5	Bell [6]
	Malawi	1998-2014	blood	2,054	Feasey [7]
	Uganda	2003-2004	blood	5	Bachou [8]
	Kenya	2004-2007	blood	144	Mutai [9]
	Tanzania	2008-2016	blood	129	Msemo [10]
	Tanzania	2009-2010	blood	46	Thriemer [11]
	Zambia*	2010-2012	blood	86	Hendriksen* [12]
	Madagascar	2011-2013	blood	8	Al-Emran [13]
	Tanzania	2011-2013	blood	9	Al-Emran [13]
	Kenya	2011-2013	blood	54	Al-Emran [13]
	Madagascar	2011-2013	blood	9	Marks [14]
	Tanzania	2011-2013	blood	9	Marks [14]
	Kenya	2012-2013	blood	54	Marks [14]
	Tanzania	2012-2013	blood	7	Onken [15]
	Ethiopia	2012-2014	blood	3	Marks [14]
	Tanzania	2013	blood	17	Mahende [16]

	Ethiopia	2014	blood	8	Wasihun [17]
	Ethiopia	2016	blood	14	Admassu [18]
Middle Africa	Democratic Republic of the Congo	1989-1990	blood	2	Bahwere [19]
	Democratic Republic of the Congo*	2004-2005	blood	11	Muyembe-Tamfum* [20]
	Democratic Republic of the Congo	2007-2011	blood	201	Lunguya [21]
	Democratic Republic of the Congo	2007-2017	blood	469	Tack [22]
	Democratic Republic of the Congo	2011-2012	blood	18	Phoba [23]
	Democratic Republic of the Congo*	2011-2014	blood	164	Kalonji* [24]
Northern Africa	Egypt	2002	blood	90	Srikantiah [25]
	Egypt	2002-2007	blood	654	Rahman [26]
	Algeria	2005-2008	blood	178	Bouzenoune [27]
	Egypt	2007-2009	blood	52	Hammad [28]
Southern Africa	South Africa	1979-1985	blood	3,313	Coovadia [29]
	South Africa	2012-2014	blood	2	Marks [14]
Western Africa	Nigeria	1980-1987	blood	14	Ako-Nai [30]
	Ivory Coast	1991	blood	5	Vugia [31]
	Nigeria	1993-1994	blood	4	Ibe [32]
	Ghana	1993-1994	blood	6	Wilkens [33]
	Senegal	1995-2002	blood	65	Lefebvre [34]
	Nigeria	1997-1998	blood	68	Akinyemi [35]
	Nigeria	2000-2001	blood	41	Akinyemi [36]
	Nigeria	2003-2004	blood	3	Samuel [37]

	Nigeria	2004-2005	blood	7	Fashae [38]
	Ghana	2007-2009	blood	17	Nielsen [39]
	Guinea-Bissau	2010	blood	3	Isendahl [40]
	Ghana	2010-2012	blood	30	Marks [14]
	Burkina Faso	2011-2013	blood	15	Al-Emran [13]
	Guinea-Bissau	2011-2013	blood	3	Al-Emran [13]
	Senegal	2011-2013	blood	7	Al-Emran [13]
	Guinea-Bissau	2011-2013	blood	3	Marks [14]
	Senegal	2011-2013	blood	7	Marks [14]
	Burkina Faso	2012-2013	blood	18	Marks [14]
	Burkina Faso	2012-2013	blood	12	Maltha [41]
**Americas**					
South America	Peru	2008-2012	blood	33	Garcia [42]
	Peru	2008-2013	blood	10	Silva [43]
**Asia**					
Central Asia	Uzbekistan	2002-2003	blood	92	Srikantiah [44]
	Uzbekistan	2002-2007	blood	123	Rahman [45]
Eastern Asia	Taiwan	1982-1995	blood, bone marrow	42	Chiu [46]
	China	1988-1989	blood	142	Zhang [47]
	Taiwan	1991-1996	blood	3	Chen [48]
	China	2001-2002	blood	15	Ochiai [49]
	China	2002-2007	blood	25	Wu [50]
South-eastern Asia	Vietnam	1972-1973	blood	163	Brown [51]
	Thailand	1977-1984	blood	163	Thisyakorn [52]
	Vietnam	1990-1993	blood, bone marrow	39	Nguyen [53]
	Philippines	1994-1997	blood	422	Abucejo [54]
	Vietnam	1995-1997	blood, bone marrow	89	Wain [55]

	Indonesia	1997-2000	blood	13	Punjabi [56]
	Myanmar	1998-1999	blood	28	Shwe [57]
	Thailand*	1999	blood	5	Swaddiwudhipong* [58]
	Vietnam	2000-2002	blood	58	Olsen [59]
	Laos	2000-2004	blood	246	Phetsouvanh [60]
	Laos	2001-2003	blood	50	Phongmany [61]
	Indonesia	2002-2003	blood	131	Ochiai [49]
	Vietnam	2002-2003	blood	18	Ochiai [49]
	Vietnam	2004-2005	blood	263	Dolecek [62]
	Cambodia	2006-2009	blood	41	Kasper [63]
	Cambodia	2007-2011	blood	148	Emary [64]
	Cambodia	2007-2011	blood	20	Vlieghe [65]
	Indonesia	2008	blood	8	Yanagi [66]
	Cambodia	2008-2015	blood	64	Kuijpers [67]
	Cambodia	2009-2010	blood	22	Chheng [68]
	Thailand*	2009-2011	blood	368	Limpitikul* [69]
	Indonesia	2011-2015	blood	93	Hardjo Lugito [70]
Southern Asia	India	1978-1987	blood	4,005	Talawadekar [71]
	Bangladesh	1979-1980	blood	58	Stoll [72]
	India*	1985-1985	blood	12	Jain* [73]
	India	1989-1990	blood, bone marrow	216	Anand [74]
	India*	1989-1990	blood, bone marrow	103	Arora* [75]
	India	1989-1990	blood, bone marrow	48	Koul [76]
	India*	1989-1991	blood	50	Haldar* [77]
	India	1989-1991	blood	283	Jesudason [78]
	Bangladesh	1989-2002	blood	3,927	Rahman [45]
	India	1990	blood	25	Agarwal [79]

	India	1990	blood	158	Dar [80]
	India*	1990	blood	50	Mishra* [81]
	Pakistan	1990	blood	109	Rathore [82]
	India	1990-1991	blood	102	Rao [83]
	India	1990-1992	blood	133	Dutta [84]
	India	1990-1992	blood	221	Rasaily [85]
	India	1990-2000	blood	336	Saha [86]
	India	1991	blood	24	Gupta [87]
	India	1991	blood	190	Rathish [88]
	India	1991-2001	blood	421	Mandal [89]
	India	1991-2003	blood	464	Mandal [90]
	Pakistan	1993	blood	12	Sultana [91]
	India	1993-1994	blood	30	Maheshwari [92]
	India	1993-2003	blood	472	Renuka [93]
	Pakistan	1994	blood	62	Mirza [94]
	Bangladesh	1994-1995	blood	78	Hermans [95]
	India	1995-1999	blood	261	Kumar [96]
	India	1995-2000	blood	94	Kapil [97]
	Bangladesh	1996-1998	blood	151	Zahurul Haque Asna [98]
	Pakistan	1996-2003	blood	477	Butt [99]
	India	1997-1998	blood	715	Das [100]
	India	1997-2001	blood	436	Gautam [101]
	India	1998-2012	blood	164	Das [102]
	India	1999-2001	blood	254	Bhattacharya [103]
	India	1999-2001	blood	44	Chowta [104]
	India	1999-2004	blood	629	Mohanty [105]
	India*	2000	blood	93	Misra* [106]
	India	2000	blood	48	Sekar [107]
	Nepal	2000-2003	blood	76	Guha [108]

	Nepal	2000-2004	blood	132	Khanal [109]
	Pakistan	2001-2002	blood	41	Anjum [110]
	Nepal	2001-2002	blood	55	Sharma [111]
	India	2001-2003	blood	60	Kadhiravan [112]
	India	2001-2003	blood	304	Walia [113]
	India	2001-2004	blood	60	Lakshmi [114]
	Pakistan	2001-2006	blood	3,671	Hasan [115]
	Nepal*	2002	blood	60	Lewis* [116]
	India	2002-2003	blood	157	Madhulika [117]
	India	2002-2003	blood	43	Mandal [118]
	India	2002-2003	blood	70	Ray [119]
	Nepal	2002-2004	blood	1,039	Malla [120]
	Pakistan	2002-2004	blood	189	Ochiai [49]
	Nepal	2002-2004	blood	50	Sharma [121]
	India	2002-2008	blood, bone marrow	240	Arora [122]
	India	2002-2013	blood	1,905	Joshi [123]
	Nepal	2003	blood	30	Shirakawa [124]
	Bangladesh	2003-2004	blood	40	Naheed [125]
	India	2003-2004	blood	122	Ochiai [49]
	India	2003-2005	blood	195	Sen [126]
	India	2003-2006	blood	372	Holt [127]
	India	2004	blood	25	Joshi [128]
	Nepal	2004	blood	409	Maskey [129]
	India	2004-2005	blood	56	Manchanda [130]
	Nepal	2004-2005	blood	46	Mathura [131]
	Nepal	2004-2006	blood	93	Tamang [132]
	Nepal	2005	blood	78	Amatya [133]
	Pakistan	2005	blood	29	Mirza [134]

	Nepal	2005-2006	blood	53	Kelly [135]
	India	2005-2006	blood	431	Raveendran [136]
	Bangladesh	2005-2006	blood	943	Sarker [137]
	India	2005-2006	blood	71	Vidyalakshmi [138]
	India	2005-2008	blood	234	Bhattacharya [139]
	India	2005-2008	blood	80	Rai [140]
	India	2005-2009	blood	337	Menezes [141]
	Bangladesh	2005-2014	blood	5,191	Ahmed [142]
	India	2005-2016	blood	772	Sharma [143]
	Pakistan	2006-2009	blood	111	Abbasi [144]
	Nepal	2007-2008	blood	195	Prajapati [145]
	India	2007-2009	blood, bone marrow	132	Gopal [146]
	India	2007-2012	blood	101	Elumalai [147]
	India	2007-2016	blood	408	Iyer [148]
	Nepal	2008	blood	29	Acharya [149]
	Pakistan	2008-2009	blood	48	Ali [150]
	India	2008-2009	blood	128	Kumar [151]
	Nepal	2008-2009	blood	9	Pokharel [152]
	India	2008-2010	blood	257	Gupta [153]
	India	2008-2012	blood, bone marrow, pus, bile	128	Bandyopadhyay [154]
	Nepal	2008-2016	blood	198	Britto [155]
	India	2009	blood	28	Alam [156]
	Nepal	2009-2010	blood	114	Acharya [157]
	Sri Lanka	2009-2010	blood	19	Chandrasiri [158]
	India*	2009-2010	blood	18	Singla* [159]
	India	2009-2011	blood	186	Choudhary [160]
	Pakistan	2009-2011	blood	2,576	Qamar [161]

	India	2009-2011	blood	85	Shetty [162]
	India	2009-2013	blood	77	Dutta [163]
	Pakistan	2010-2011	blood	80	Afzal [164]
	India	2010-2011	blood	36	Geetha [165]
	Nepal	2010-2011	blood	47	Raza [166]
	India	2010-2012	blood	266	Jain [167]
	India	2010-2014	blood	630	Yadav [168]
	India	2011	blood	61	Vala [169]
	Nepal	2011-2012	blood	56	Chand [170]
	Pakistan	2011-2012	blood	71	Ikram [171]
	India	2011-2013	blood	16	Sania [172]
	India	2011-2017	blood	623	Makkar [173]
	Nepal	2012	blood	10	Roka [174]
	India	2012-2013	blood	17	Garg [175]
	Nepal	2012-2013	blood	48	Shrestha [176]
	India	2012-2013	blood	16	Srirangaraj [177]
	Pakistan	2012-2014	blood	155	Ali [178]
	India	2012-2014	blood	283	Bakthavatchalam [179]
	Nepal	2012-2014	blood	64	Petersiel [180]
	Pakistan	2012-2014	blood	1,979	Qamar [181]
	India	2013	blood	167	Sharvani [182]
	India	2013-2015	blood	39	Ejaz [183]
	India	2013-2015	blood	80	Misra [184]
	Bangladesh	2014	blood	181	Ali [185]
	Pakistan	2014	blood	80	Erum [186]
	Pakistan	2014-2015	blood	270	Zehra [187]
	India*	2015-2016	blood	42	Purighalla* [188]
	Nepal	2015-2017	blood	162	Bhetwal [189]
	Nepal	2016	blood	74	Khanal [190]

	Pakistan	2016-2017	blood	161	Imran [191]
	India	2016-2017	blood	49	Sucindar [192]
	Pakistan	2017	blood	323	Shaikh [193]
	Pakistan	2018-2018	blood	223	Laghari [194]
Western Asia	Lebanon	1998-2001	blood	127	Hamze [195]
	Kuwait	2002-2006	blood	136	Dimitrov [196]
	United Arab Emirates	2007-2009	blood	118	Abro [197]
**Europe**					
Southern Europe	Greece	1990-2002	blood	22	Papaevangelou [198]

* Study was of an outbreak

REFERENCES

1. Kariuki S, Gilks C, Revathi G, Hart CA. Genotypic analysis of multidrug-resistant Salmonella enterica Serovar typhi, Kenya. *Emerg Infect Dis*. 2000;6(6):649-651. doi:10.3201/eid0606.000616.

2. Dougle ML, Hendriks ER, Sanders EJ, Dorigo-Zetsma JW. Laboratory investigations in the diagnosis of septicaemia and malaria. *East Afr Med J*. 1997;74(6):353-356.

3. Walsh AL, Phiri AJ, Graham SM, Molyneux EM, Molyneux ME. Bacteremia in febrile Malawian children: clinical and microbiologic features. *Pediatr Infect Dis J*. 2000;19(4):312-318. doi:10.1097/00006454-200004000-00010.

4. Gordon MA, Walsh AL, Chaponda M, Soko D, Mbvwinji M, Molyneux ME, Gordon SB. Bacteraemia and mortality among adult medical admissions in Malawi--predominance of non-typhi salmonellae and Streptococcus pneumoniae. *J Infect*. 2001;42(1):44-49. doi:10.1053/jinf.2000.0779.

5. Issack MI. Epidemiology of typhoid fever in Mauritius. *J Travel Med*. 2005;12(5):270-274. doi:10.2310/7060.2005.12506.

6. Bell M, Archibald LK, Nwanyanwu O, Dobbie H, Tokars J, Kazembe PN, Reller LB, Jarvis WR. Seasonal variation in the etiology of bloodstream infections in a febrile inpatient population in a developing country. *Int J Infect Dis*. 2001;5(2):63-69. doi:10.1016/s1201-9712(01)90027-x.

7. Feasey NA, Gaskell K, Wong V, Msefula C, Selemani G, Kumwenda S, Allain TJ, Mallewa J, Kennedy N, Bennett A, Nyirongo JO, Nyondo PA, Zulu MD, Parkhill J, Dougan G, Gordon MA, Heyderman RS. Rapid emergence of multidrug resistant, H58-lineage Salmonella typhi in Blantyre, Malawi. *PLoS Negl Trop Dis*. 2015;9(4):e0003748. doi:10.1371/journal.pntd.0003748.

8. Bachou H, Tylleskär T, Kaddu-Mulindwa DH, Tumwine JK. Bacteraemia among severely malnourished children infected and uninfected with the human immunodeficiency virus-1 in Kampala, Uganda. *BMC Infect Dis*. 2006;6:160. doi:10.1186/1471-2334-6-160.

9. Mutai WC, Muigai AWT, Waiyaki P, Kariuki S. Multi-drug resistant Salmonella enterica serovar Typhi isolates with reduced susceptibility to ciprofloxacin in Kenya. *BMC Microbiol*. 2018;18(1):187. doi:10.1186/s12866-018-1332-3.

10. Msemo OA, Mbwana J, Mahende C, Malabeja A, Gesase S, Crump JA, Dekker D, Lusingu JPA. Epidemiology and antimicrobial susceptibility of Salmonella enterica bloodstream isolates among febrile children in a rural district in northeastern Tanzania: A cross-sectional study. *Clin Infect Dis*. 2019;68(Suppl 2):S177-S182. doi:10.1093/cid/ciy1126.

11. Thriemer K, Ley B, Ame S, von Seidlein L, Pak GD, Chang NY, Hashim R, Schmied WH, Busch CJ-L, Nixon S, Morrissey A, Puri MK, Ali M, Ochiai RL, Wierzba T, Jiddawi MS, Clemens JD, Ali SM, Deen JL. The burden of invasive bacterial infections in Pemba, Zanzibar. *PloS One*. 2012;7(2):e30350. doi:10.1371/journal.pone.0030350.

12. Hendriksen RS, Leekitcharoenphon P, Lukjancenko O, Lukwesa-Musyani C, Tambatamba B, Mwaba J, Kalonda A, Nakazwe R, Kwenda G, Jensen JD, Svendsen CA, Dittmann KK, Kaas RS, Cavaco LM, Aarestrup FM, Hasman H, Mwansa JCL. Genomic signature of multidrug-resistant Salmonella enterica serovar typhi isolates related to a massive outbreak in Zambia between 2010 and 2012. *J Clin Microbiol*. 2015;53(1):262-272. doi:10.1128/JCM.02026-14.

13. Al-Emran HM, Eibach D, Krumkamp R, Ali M, Baker S, Biggs HM, Bjerregaard-Andersen M, Breiman RF, Clemens JD, Crump JA, Cruz Espinoza LM, Deerin J, Dekker DM, Gassama Sow A, Hertz JT, Im J, Ibrango S, von Kalckreuth V, Kabore

LP, Konings F, Løfberg SV, Meyer CG, Mintz ED, Montgomery JM, Olack B, Pak GD, Panzner U, Park SE, Razafindrabe JLT, Rabezanahary H, Rakotondrainiarivelo JP, Rakotozandrindrainy R, Raminosoa TM, Schütt-Gerowitt H, Sampo E, Soura AB, Tall A, Warren M, Wierzba TF, May J, Marks F. A multicountry molecular analysis of Salmonella enterica serovar Typhi with reduced susceptibility to ciprofloxacin in Sub-Saharan Africa. *Clin Infect Dis*. 2016;62 Suppl 1:S42-46. doi:10.1093/cid/civ788.

14. Marks F, von Kalckreuth V, Aaby P, Adu-Sarkodie Y, El Tayeb MA, Ali M, Aseffa A, Baker S, Biggs HM, Bjerregaard-Andersen M, Breiman RF, Campbell JI, Cosmas L, Crump JA, Espinoza LMC, Deerin JF, Dekker DM, Fields BS, Gasmelseed N, Hertz JT, Van Minh Hoang N, Im J, Jaeger A, Jeon HJ, Kabore LP, Keddy KH, Konings F, Krumkamp R, Ley B, Løfberg SV, May J, Meyer CG, Mintz ED, Montgomery JM, Niang AA, Nichols C, Olack B, Pak GD, Panzner U, Park JK, Park SE, Rabezanahary H, Rakotozandrindrainy R, Raminosoa TM, Razafindrabe TJL, Sampo E, Schütt-Gerowitt H, Sow AG, Sarpong N, Seo HJ, Sooka A, Soura AB, Tall A, Teferi M, Thriemer K, Warren MR, Yeshitela B, Clemens JD, Wierzba TF. Incidence of invasive salmonella disease in sub-Saharan Africa: a multicentre population-based surveillance study. *Lancet Glob Health*. 2017;5(3):e310-e323. doi:10.1016/S2214-109X(17)30022-0.

15. Onken A, Said AK, Jørstad M, Jenum PA, Blomberg B. Prevalence and antimicrobial resistance of microbes causing bloodstream infections in Unguja, Zanzibar. *PloS One*. 2015;10(12):e0145632. doi:10.1371/journal.pone.0145632.

16. Mahende C, Ngasala B, Lusingu J, Butichi A, Lushino P, Lemnge M, Mmbando B, Premji Z. Bloodstream bacterial infection among outpatient children with acute febrile illness in north-eastern Tanzania. *BMC Res Notes*. 2015;8:289. doi:10.1186/s13104-015-1178-9.

17. Wasihun AG, Wlekidan LN, Gebremariam SA, Dejene TA, Welderufael AL, Haile TD, Muthupandian S. Bacteriological profile and antimicrobial susceptibility patterns of blood culture isolates among febrile patients in Mekelle Hospital, Northern Ethiopia. *SpringerPlus*. 2015;4:314. doi:10.1186/s40064-015-1056-x.

18. Admassu D, Egata G, Teklemariam Z. Prevalence and antimicrobial susceptibility pattern of Salmonella enterica serovar Typhi and Salmonella enterica serovar Paratyphi among febrile patients at Karamara Hospital, Jigjiga, eastern Ethiopia. *SAGE Open Med*. 2019;7:2050312119837854. doi:10.1177/2050312119837854.

19. Bahwere P, Levy J, Hennart P, Donnen P, Lomoyo W, Dramaix-Wilmet M, Butzler JP, De Mol P. Community-acquired bacteremia among hospitalized children in rural central Africa. *Int J Infect Dis*. 2001;5(4):180-188. doi:10.1016/s1201-9712(01)90067-0.

20. Muyembe-Tamfum JJ, Veyi J, Kaswa M, Lunguya O, Verhaegen J, Boelaert M. An outbreak of peritonitis caused by multidrug-resistant Salmonella Typhi in Kinshasa, Democratic Republic of Congo. *Travel Med Infect Dis*. 2009;7(1):40-43. doi:10.1016/j.tmaid.2008.12.006.

21. Lunguya O, Lejon V, Phoba M-F, Bertrand S, Vanhoof R, Verhaegen J, Smith AM, Keddy KH, Muyembe-Tamfum J-J, Jacobs J. Salmonella Typhi in the democratic republic of the congo: fluoroquinolone decreased susceptibility on the rise. *PLoS Negl Trop Dis*. 2012;6(11):e1921. doi:10.1371/journal.pntd.0001921.

22. Tack B, Phoba M-F, Van Puyvelde S, Kalonji LM, Hardy L, Barbé B, Van der Sande MAB, Monsieurs E, Deborggraeve S, Lunguya O, Jacobs J. Salmonella Typhi from blood cultures in the Democratic Republic of the Congo: a 10-Year surveillance. *Clin Infect Dis*. 2019;68(Suppl 2):S130-S137. doi:10.1093/cid/ciy1116.

23. Phoba M-F, De Boeck H, Ifeka BB, Dawili J, Lunguya O, Vanhoof R, Muyembe J-J, Van Geet C, Bertrand S, Jacobs J. Epidemic increase in Salmonella bloodstream infection in children, Bwamanda, the Democratic Republic of Congo. *Eur J Clin Microbiol Infect Dis*. 2014;33(1):79-87. doi:10.1007/s10096-013-1931-8.

24. Kalonji LM, Post A, Phoba M-F, Falay D, Ngbonda D, Muyembe J-J, Bertrand S, Ceyssens P-J, Mattheus W, Verhaegen J, Barbé B, Kuijpers L, Van Geet C, Lunguya O, Jacobs J. Invasive Salmonella infections at multiple surveillance sites in the Democratic Republic of the Congo, 2011-2014. *Clin Infect Dis*. 2015;61 Suppl 4:S346-353. doi:10.1093/cid/civ713.

25. Srikantiah P, Girgis FY, Luby SP, Jennings G, Wasfy MO, Crump JA, Hoekstra RM, Anwer M, Mahoney FJ. Population-based surveillance of typhoid fever in Egypt. *Am J Trop Med Hyg*. 2006;74(1):114-119.

26. Rahman BA, Wasfy MO, Maksoud MA, Hanna N, Dueger E, House B. Multi-drug resistance and reduced susceptibility to ciprofloxacin among Salmonella enterica serovar Typhi isolates from the Middle East and Central Asia. *New Microbes New Infect*. 2014;2(4):88-92. doi:10.1002/nmi2.46.

27. Bouzenoune F, Kellab Debbih K, Boudersa F, Kouhil S, Nezzar N. [Antibiotic susceptibility of Salmonella enterica serovar Typhi isolated from blood cultures at the Ain M'lila hospital (Algeria), between 2005 and 2008]. *Med Mal Infect*. 2011;41(4):181-185. doi:10.1016/j.medmal.2010.09.011.

28. Hammad OM, Hifnawy T, Omran D, El Tantawi MA, Girgis NI. Ceftriaxone versus chloramphenicol for treatment of acute typhoid fever. *Life Sci J*. 2011;8(2):100-105.

29. Coovadia YM, Van den Ende J. Chloramphenicol-resistant Salmonella typhi in Durban, South Africa. *Trop Geogr Med*. 1987;39(1):64-66.

30. Ako-Nai AK, Taiwo O, Ebri A, Adeniran MO. Bacterial isolates involved in cases of septicaemia in a Nigerian hospital. *East Afr Med J*. 1990;67(6):407-412.

31. Vugia DJ, Kiehlbauch JA, Yeboue K, N'Gbichi JM, Lacina D, Maran M, Gondo M, Kouadio K, Kadio A, Lucas SB, Kestens L, Crawford JT, Wells JG, Brattegaard K, De Cock KM, Griffin PM. Pathogens and Predictors of Fatal Septicemia Associated with Human Immunodeficiency Virus Infection in Ivory Coast, West Africa. *J Infect Dis*. 1993;168(3):564-570. doi:10.1093/infdis/168.3.564.

32. Ibe SN, Wariso BA. Drug susceptibility profile of Salmonella typhi blood isolates in Port Harcourt, Nigeria. *West Afr J Med*. 1996;15(4):219-222.

33. Wilkens J, Newman MJ, Commey JO, Seifert H. Salmonella bloodstream infection in Ghanaian children. *Clin Microbiol Infect*. 1997;3(6):616-620. doi:10.1111/j.1469-0691.1997.tb00467.x.

34. Lefebvre N, Gning SB, Nabeth P, Ka S, Ba-Fall K, Rique M, Sane M, Chevalier B, Mbaye PS, Debonne JM. [Clinical and laboratory features of typhoid fever in Senegal. A 70-case study]. *Med Trop Rev Corps Sante Colon*. 2005;65(6):543-548.

35. Akinyemi KO, Coker AO, Olukoya DK, Oyefolu AO, Amorighoye EP, Omonigbehin EO. Prevalence of multi-drug resistant Salmonella typhi among clinically diagnosed typhoid fever patients in Lagos, Nigeria. *J Biosci*. 2000;55(5-6):489-493. doi:10.1515/znc-2000-5-630.

36. Akinyemi KO, Smith SI, Oyefolu AOB, Coker AO. Multidrug resistance in Salmonella enterica serovar typhi isolated from patients with typhoid fever complications in Lagos, Nigeria. *Public Health*. 2005;119(4):321-327. doi:10.1016/j.puhe.2004.04.009.

37. Samuel SO, Fadeyi A, Akanbi AA, Ameen NB, Nwabuisi C, Onile BA. Bacterial isolates of blood cultures in patients with suspected septicaemia in Ilorin, Nigeria. *Afr J Med Med Sci*. 2006;35(2):137-141.

38. Fashae K, Ogunsola F, Aarestrup FM, Hendriksen RS. Antimicrobial susceptibility and serovars of Salmonella from chickens and humans in Ibadan, Nigeria. *J Infect Dev Ctries*. 2010;4(8):484-494. doi:10.3855/jidc.909.

39. Nielsen MV, Sarpong N, Krumkamp R, Dekker D, Loag W, Amemasor S, Agyekum A, Marks F, Huenger F, Krefis AC, Hagen RM, Adu-Sarkodie Y, May J, Schwarz NG. Incidence and characteristics of bacteremia among children in rural Ghana. *PloS One*. 2012;7(9):e44063. doi:10.1371/journal.pone.0044063.

40. Isendahl J, Manjuba C, Rodrigues A, Xu W, Henriques-Normark B, Giske CG, Nauclér P. Prevalence of community-acquired bacteraemia in Guinea-Bissau: an observational study. *BMC Infect Dis*. 2014;14:3859. doi:10.1186/s12879-014-0715-9.

41. Maltha J, Guiraud I, Kaboré B, Lompo P, Ley B, Bottieau E, Van Geet C, Tinto H, Jacobs J. Frequency of severe malaria and invasive bacterial infections among children admitted to a rural hospital in Burkina Faso. *PloS One*. 2014;9(2):e89103. doi:10.1371/journal.pone.0089103.

42. García C, Lejon V, Horna G, Astocondor L, Vanhoof R, Bertrand S, Jacobs J. Intermediate susceptibility to ciprofloxacin among Salmonella enterica serovar Typhi isolates in Lima, Peru. *J Clin Microbiol*. 2014;52(3):968-970. doi:10.1128/JCM.02663-13.

43. Silva C, Betancor L, García C, Astocondor L, Hinostroza N, Bisio J, Rivera J, Perezgasga L, Pérez Escanda V, Yim L, Jacobs J, García-Del Portillo F, SalmoIber CYTED Network, Chabalgoity JA, Puente JL. Characterization of Salmonella enterica

isolates causing bacteremia in Lima, Peru, using multiple typing methods. *PloS One*. 2017;12(12):e0189946. doi:10.1371/journal.pone.0189946.

44. Srikantiah P, Vafokulov S, Luby SP, Ishmail T, Earhart K, Khodjaev N, Jennings G, Crump JA, Mahoney FJ. Epidemiology and risk factors for endemic typhoid fever in Uzbekistan. *Trop Med Int Health*. 2007;12(7):838-847. doi:10.1111/j.1365-3156.2007.01853.x.

45. Rahman M, Siddique AK, Shoma S, Rashid H, Salam MA, Ahmed QS, Nair GB, Breiman RF. Emergence of multidrug-resistant Salmonella enterica serotype Typhi with decreased ciprofloxacin susceptibility in Bangladesh. *Epidemiol Infect*. 2006;134(2):433-438. doi:10.1017/S0950268805004759.

46. Chiu CH, Tsai JR, Ou JT, Lin TY. Typhoid fever in children: a fourteen-year experience. *Acta Paediatr Taiwan*. 2000;41(1):28-32.

47. Zhang L. [Mechanism of multiresistant Salmonella typhi]. *Zhonghua Yi Xue Za Zhi*. 1991;71(6):314-317, 24.

48. Chen YH, Chen TP, Tsai JJ, Hwang KP, Lu PL, Cheng HH, Peng CF. Epidemiological study of human salmonellosis during 1991-1996 in southern Taiwan. *Kaohsiung J Med Sci*. 1999;15(3):127-136.

49. Ochiai RL, Acosta CJ, Danovaro-Holliday MC, Baiqing D, Bhattacharya SK, Agtini MD, Bhutta ZA, Canh DG, Ali M, Shin S, Wain J, Page A-L, Albert MJ, Farrar J, Abu-Elyazeed R, Pang T, Galindo CM, von Seidlein L, Clemens JD, Domi Typhoid Study Group. A study of typhoid fever in five Asian countries: disease burden and implications for controls. *Bull World Health Organ*. 2008;86(4):260-268. doi:10.2471/blt.06.039818.

50. Wu W, Wang H, Lu J, Wu J, Chen M, Xu Y, Lu Y. Genetic diversity of Salmonella enteric serovar typhi and paratyphi in Shenzhen, China from 2002 through 2007. *BMC Microbiol*. 2010;10:32. doi:10.1186/1471-2180-10-32.

51. Brown JD, Duong Hong M o, Rhoades ER. Chloramphenicol-resistant Salmonella typhi in Saigon. *J Am Med Assoc*. 1975;231(2):162-166.

52. Thisyakorn U, Mansuwan P, Taylor DN. Typhoid and paratyphoid fever in 192 hospitalized children in Thailand. *Am J Dis Child*. 1987;141(8):862-865. doi:10.1001/archpedi.1987.04460080048025.

53. Nguyen TA, Ha Ba K, Nguyen TD. [Typhoid fever in South Vietnam, 1990-1993]. *Bull Soc Pathol Exot*. 1993;86(5 Pt 2):476-478.

54. Abucejo PE, Capeding MR, Lupisan SP, Arcay J, Sombrero LT, Ruutu P, Herva E. Blood culture confirmed typhoid fever in a provincial hospital in the Philippines. *Southeast Asian J Trop Med Public Health*. 2001;32(3):531-536.

55. Wain J, Pham VB, Ha V, Nguyen NM, To SD, Walsh AL, Parry CM, Hasserjian RP, HoHo VA, Tran TH, Farrar J, White NJ, Day NP. Quantitation of bacteria in bone marrow from patients with typhoid fever: relationship between counts and clinical features. *J Clin Microbiol*. 2001;39(4):1571-1576. doi:10.1128/JCM.39.4.1571-1576.2001.

56. Punjabi NH, Taylor WRJ, Murphy GS, Purwaningsih S, Picarima H, Sisson J, Olson JG, Baso S, Wangsasaputra F, Lesmana M, Oyofo BA, Simanjuntak CH, Subekti D, Corwin AL, Richie TL. Etiology of acute, non-malaria, febrile illnesses in Jayapura, northeastern Papua, Indonesia. *Am J Trop Med Hyg*. 2012;86(1):46-51. doi:10.4269/ajtmh.2012.10-0497.

57. Shwe TN, Nyein MM, Yi W, Mon A. Blood culture isolates from children admitted to Medical Unit III, Yangon Children's Hospital, 1998. *Southeast Asian J Trop Med Public Health*. 2002;33(4):764-771.

58. Swaddiwudhipong W, Kanlayanaphotporn J. A common-source water-borne outbreak of multidrug-resistant typhoid fever in a rural Thai community. *J Med Assoc Thai*. 2001;84(11):1513-1517.

59. Olsen SJ, Pruckler J, Bibb W, Nguyen TMT, Tran MT, Nguyen TM, Sivapalasingam S, Gupta A, Phan TP, Nguyen TC, Nguyen VC, Phung DC, Mintz ED. Evaluation of rapid diagnostic tests for typhoid fever. *J Clin Microbiol*. 2004;42(5):1885-1889. doi:10.1128/jcm.42.5.1885-1889.2004.

60. Phetsouvanh R, Phongmany S, Soukaloun D, Rasachak B, Soukhaseum V, Soukhaseum S, Frichithavong K, Khounnorath S, Pengdee B, Phiasakha K, Chu V, Luangxay K, Rattanavong S, Sisouk K, Keolouangkot V, Mayxay M, Ramsay A, Blacksell SD, Campbell J, Martinez-Aussel B, Heuanvongsy M, Bounxouei B, Thammavong C, Syhavong B, Strobel M, Peacock SJ, White NJ, Newton PN. Causes of community-acquired bacteremia and patterns of antimicrobial resistance in Vientiane, Laos. *Am J Trop Med Hyg*. 2006;75(5):978-985. doi:10.4269/ajtmh.2006.75.978.

61. Phongmany S, Phetsouvanh R, Sisouphone S, Darasavath C, Vongphachane P, Rattanavong O, Mayxay M, Ramsay AC, Blacksell SD, Thammavong C, Syhavong B, White NJ, Newton PN. A randomized comparison of oral chloramphenicol versus ofloxacin in the treatment of uncomplicated typhoid fever in Laos. *Trans R Soc Trop Med Hyg*. 2005;99(6):451-458. doi:10.1016/j.trstmh.2004.08.007.

62. Dolecek C, Tran TPL, Nguyen NR, Le TP, Ha V, Phung QT, Doan CD, Nguyen TBB, Duong TL, Luong BH, Nguyen TB, Nguyen TAH, Pham ND, Mai NL, Phan VBB, Vo

AH, Nguyen VMH, Tran TTN, Tran TC, Schultsz C, Dunstan SJ, Stepniewska K, Campbell JI, To SD, Basnyat B, Nguyen VVC, Nguyen VS, Nguyen TC, Tran TH, Farrar J. A multi-center randomised controlled trial of gatifloxacin versus azithromycin for the treatment of uncomplicated typhoid fever in children and adults in Vietnam. *PloS One*. 2008;3(5):e2188. doi:10.1371/journal.pone.0002188.

63. Kasper MR, Sokhal B, Blair PJ, Wierzba TF, Putnam SD. Emergence of multidrug-resistant Salmonella enterica serovar Typhi with reduced susceptibility to fluoroquinolones in Cambodia. *Diagn Microbiol Infect Dis*. 2010;66(2):207-209. doi:10.1016/j.diagmicrobio.2009.09.002.

64. Emary K, Moore CE, Chanpheaktra N, An KP, Chheng K, Sona S, Duy PT, Nga TVT, Wuthiekanun V, Amornchai P, Kumar V, Wijedoru L, Stoesser NE, Carter MJ, Baker S, Day NPJ, Parry CM. Enteric fever in Cambodian children is dominated by multidrug-resistant H58 Salmonella enterica serovar Typhi with intermediate susceptibility to ciprofloxacin. *Trans R Soc Trop Med Hyg*. 2012;106(12):718-724. doi:10.1016/j.trstmh.2012.08.007.

65. Vlieghe ER, Phe T, De Smet B, Veng CH, Kham C, Bertrand S, Vanhoof R, Lynen L, Peetermans WE, Jacobs JA. Azithromycin and ciprofloxacin resistance in Salmonella bloodstream infections in Cambodian adults. *PLoS Negl Trop Dis*. 2012;6(12):e1933. doi:10.1371/journal.pntd.0001933.

66. Yanagi D, de Vries GC, Rahardjo D, Alimsardjono L, Wasito EB, De I, Kinoshita S, Hayashi Y, Hotta H, Osawa R, Kawabata M, Shirakawa T. Emergence of fluoroquinolone-resistant strains of Salmonella enterica in Surabaya, Indonesia. *Diagn Microbiol Infect Dis*. 2009;64(4):422-426. doi:10.1016/j.diagmicrobio.2009.04.006.

67. Kuijpers LMF, Phe T, Veng CH, Lim K, Ieng S, Kham C, Fawal N, Fabre L, Le Hello S, Vlieghe E, Weill F-X, Jacobs J, Peetermans WE. The clinical and microbiological

characteristics of enteric fever in Cambodia, 2008-2015. *PLoS Negl Trop Dis*. 2017;11(9):e0005964. doi:10.1371/journal.pntd.0005964.

68. Chheng K, Carter MJ, Emary K, Chanpheaktra N, Moore CE, Stoesser N, Putchhat H, Sona S, Reaksmey S, Kitsutani P, Sar B, van Doorn HR, Uyen NH, Van Tan L, Paris DH, Paris D, Blacksell SD, Amornchai P, Wuthiekanun V, Parry CM, Day NPJ, Kumar V. A prospective study of the causes of febrile illness requiring hospitalization in children in Cambodia. *PloS One*. 2013;8(4):e60634. doi:10.1371/journal.pone.0060634.

69. Limpitikul W, Henpraserttae N, Saksawad R, Laoprasopwattana K. Typhoid outbreak in Songkhla, Thailand 2009-2011: clinical outcomes, susceptibility patterns, and reliability of serology tests. *PloS One*. 2014;9(11):e111768. doi:10.1371/journal.pone.0111768.

70. Hardjo Lugito NP, Cucunawangsih null. Antimicrobial resistance of Salmonella enterica serovars Typhi and Paratyphi Isolates from a general hospital in Karawaci, Tangerang, Indonesia: A five-year review. *Int J Microbiol*. 2017;2017:6215136. doi:10.1155/2017/6215136.

71. Talawadekar NN, Vadher PJ, Antani DU, Kale VV, Kamat SA. Chloramphenicol resistant Salmonella species isolated between 1978 and 1987. *J Postgrad Med*. 1989;35(2):79-82.

72. Stoll BJ, Glass RI, Banu H, Alam M. Enteric fever in patients admitted to a diarrhoeal disease hospital in Bangladesh. *Trans R Soc Trop Med Hyg*. 1983;77(4):548-551. doi:10.1016/0035-9203(83)90134-7.

73. Jain S, Chitnis DS, Sham A, Rathi S, Inamdar S, Rindani GJ. Outbreak of chloramphenicol resistant typhoid fever. *Indian Pediatr*. 1987;24(3):193-197.

74. Anand AC. The anatomy of an epidemic (the final report on an epidemic of multidrug resistant enteric fever in eastern India). *Trop Gastroenterol Off J Dig Dis Found*. 1993;14(1):21-27.

75. Arora RK, Gupta A, Joshi NM, Kataria VK, Lall P, Anand AC. Multidrug resistant typhoid fever: study of an outbreak in Calcutta. *Indian Pediatr*. 1992;29(1):61-66.

76. Koul PB, Murali MV, Sharma PP, Ghai OP, Ramchandran VG, Talwar V. Multi drug resistant Salmonella typhi infection: clinical profile and therapy. *Indian Pediatr*. 1991;28(4):357-361.

77. Haldar KK, Basak S, Chakraborty AK, Das S. Transferable drug resistance in Salmonella typhi strains isolated from an outbreak at Calcutta in the recent past. *J Indian Med Assoc*. 1995;93(8):299-300, 315.

78. Jesudason MV, John TJ. Plasmid mediated multidrug resistance in Salmonella typhi. *Indian J Med Res*. 1992;95:66-67.

79. Agarwal S, Madhu SV, Guleria JS, Talwar V. The problem of emerging chloramphenicol resistance in typhoid fever--a preliminary report. *J Assoc Physicians India*. 1991;39(6):443-444.

80. Dar L, Gupta BL, Rattan A, Bhujwala RA, Shriniwas null. Multidrug resistant Salmonella typhi in Delhi. *Indian J Pediatr*. 1992;59(2):221-224. doi:10.1007/bf02759988.

81. Mishra S, Patwari AK, Anand VK, Pillai PK, Aneja S, Chandra J, Sharma D. Multidrug resistant typhoid fever: therapeutic considerations. *Indian Pediatr*. 1992;29(4):443-448.

82. Rathore MH, Bux D, Hasan M. Multidrug-resistant Salmonella Typhi in Pakistani children: clinical features and treatment. *South Med J*. 1996;89(2):235-237. doi:10.1097/00007611-199602000-00017.

83. Rao PS, Rajashekar V, Varghese GK, Shivananda PG. Emergence of multidrug-resistant Salmonella typhi in rural southern India. *Am J Trop Med Hyg*. 1993;48(1):108-111. doi:10.4269/ajtmh.1993.48.108.

84. Dutta P, Rasaily R, Saha MR, Mitra U, Manna B, Chakraborty S, Mukherjee A. Randomized clinical trial of furazolidone for typhoid fever in children. *Scand J Gastroenterol*. 1993;28(2):168-172. doi:10.3109/00365529309096065.

85. Rasaily R, Dutta P, Saha MR, Mitra U, Lahiri M, Pal SC. Multi-drug resistant typhoid fever in hospitalised children. Clinical, bacteriological and epidemiological profiles. *Eur J Epidemiol*. 1994;10(1):41-46. doi:10.1007/bf01717450.

86. Saha MR, Dutta P, Niyogi SK, Dutta S, Mitra U, Ramamurthy T, Manna B, Bhattacharya SK. Decreasing trend in the occurrence of Salmonella enterica serotype Typhi amongst hospitalised children in Kolkata, India during 1990-2000. *Indian J Med Res*. 2002;115:46-48.

87. Gupta S, Meena HS. Changing profile of enteric fever in summer 91. *J Assoc Physicians India*. 1992;40(11):726-729.

88. Rathish KC, Chandrashekar MR, Nagesha CN. Multidrug resistant Salmonella Typhi in Bangalore, south India. *Indian J Med Sci*. 1994;48(4):85-88.

89. Mandal S, Mandal MD, Pal NK. Ofloxacin minimum inhibitory concentration versus disk diffusion zone diameter for Salmonella enterica serovar Typhi isolates: problems in the detection of ofloxacin resistance. *Jpn J Infect Dis*. 2003;56(5-6):210-212.

90. Mandal S, Mandal MD, Pal NK. In vitro activity of gentamicin and amikacin against Salmonella enterica serovar Typhi: a search for a treatment regimen for typhoid fever. *East Mediterr Health J*. 2009;15(2):264-268.

91. Sultana K, Ashiq BM, Ikram N. Evaluation of antibiotic resistance in clinical isolates of Salmonella typhi from Islamabad. *Pak J Zool*. 1995;27(2):185-187.

92. Maheshwari VD, Agarwal SK. Present status of drug resistance in cases of enteric fever in Rajasthan. *J Assoc Physicians India*. 1996;44(9):618-619.

93. Renuka K, Kapil A, Kabra SK, Wig N, Das BK, Prasad VVSP, Chaudhry R, Seth P. Reduced susceptibility to ciprofloxacin and gyra gene mutation in North Indian strains of Salmonella enterica serotype Typhi and serotype Paratyphi A. *Microb Drug Resist*. 2004;10(2):146-153. doi:10.1089/1076629041310028.

94. Mirza SH, Beeching NJ, Hart CA. The prevalence and clinical features of multi-drug resistant Salmonella typhi infections in Baluchistan, Pakistan. *Ann Trop Med Parasitol*. 1995;89(5):515-519. doi:10.1080/00034983.1995.11812984.

95. Hermans PW, Saha SK, van Leeuwen WJ, Verbrugh HA, van Belkum A, Goessens WH. Molecular typing of Salmonella typhi strains from Dhaka (Bangladesh) and development of DNA probes identifying plasmid-encoded multidrug-resistant isolates. *J Clin Microbiol*. 1996;34(6):1373-1379.

96. Kumar R, Aneja KR, Roy P, Sharma M, Gupta R, Ram S. Evaluation of minimum inhibitory concentration of quinolones and third generation cephalosporins to Salmonella typhi isolates. *Indian J Med Sci*. 2002;56(1):1-8.

97. Kapil A, Renuka null, Das B. Nalidixic acid susceptibility test to screen ciprofloxacin resistance in Salmonella typhi. *Indian J Med Res*. 2002;115:49-54.

98. Zahurul Haque Asna SM, Ashraful Haq J. Decrease of antibiotic resistance in Salmonella typhi isolated from patients attending hospitals of Dhaka City over a 3 year period. *Int J Antimicrob Agents*. 2000;16(3):249-251. doi:10.1016/s0924-8579(00)00230-2.

99. Butt T, Ahmad RN, Salman M, Kazmi SY. Changing trends in drug resistance among typhoid salmonellae in Rawalpindi, Pakistan. *East Mediterr Health J*. 2005;11(5-6):1038-1044.

100. Das U, Bhattacharya SS. Multidrug resistant Salmonella typhi in Rourkela, Orissa. *Indian J Pathol Microbiol*. 2000;43(2):135-138.

101. Gautam V, Gupta NK, Chaudhary U, Arora DR. Sensitivity pattern of Salmonella serotypes in Northern India. *Braz J Infect Dis Off Publ Braz Soc Infect Dis*. 2002;6(6):281-287. doi:10.1590/s1413-86702002000600003.

102. Das S, Samajpati S, Ray U, Roy I, Dutta S. Antimicrobial resistance and molecular subtypes of Salmonella enterica serovar Typhi isolates from Kolkata, India over a 15 years period 1998-2012. *Int J Med Microbiol*. 2017;307(1):28-36. doi:10.1016/j.ijmm.2016.11.006.

103. Bhattacharya SS, Das U. A steady decrease in occurrence of Salmonella typhi infection in Rourkela, Orissa. *Indian J Pathol Microbiol*. 2003;46(3):498-500.

104. Chowta MN, Chowta NK. Study of clinical profile and antibiotic response in typhoid fever. *Indian J Med Microbiol*. 2005;23(2):125-127. doi:10.4103/0255-0857.16054.

105. Mohanty S, Renuka K, Sood S, Das BK, Kapil A. Antibiogram pattern and seasonality of Salmonella serotypes in a North Indian tertiary care hospital. *Epidemiol Infect*. 2006;134(5):961-966. doi:10.1017/S0950268805005844.

106. Misra RN, Bawa KS, Magu SK, Bhandari S, Nagendra A, Menon PK. Outbreak of multi-drug resistant Salmonella Typhi enteric fever in Mumbai Garrison. *Med J Armed Forces India*. 2005;61(2):148-150. doi:10.1016/S0377-1237(05)80011-5.

107. Sekar U, Srikanth P, Kindo AJ, Babu VP, Ramasubramanian V. Increase in minimum inhibitory concentration to quinolones and ceftriaxone in salmonellae causing enteric fever. *J Commun Dis*. 2003;35(3):162-169.

108. Guha S, Jalan BY, Dey S, Easow JM, Wilson G, Shivananda PG. Salmonella bacteraemia in Pokhara: emergence of antibiotic resistance. *Nepal Med Coll J*. 2005;7(1):23-25.

109. Khanal B, Sharma SK, Bhattacharya SK, Bhattarai NR, Deb M, Kanungo R. Antimicrobial susceptibility patterns of Salmonella enterica serotype typhi in eastern Nepal. *J Health Popul Nutr*. 2007;25(1):82-87.

110. Anjum P, Qureshi AH, Rafi S. Fluroquinolone resistance in typhoidal Salmonella and its detection by nalidixic acid disc diffusion. *J Pak Med Assoc*. 2004;54(6):295-301.

111. Sharma N, Koju R, Karmacharya B, Tamang M-D, Makaju R, Nepali N, Shrestha P, Adhikari D. Typhoid fever in Dhulikhel hospital, Nepal. *Kathmandu Univ Med J*. 2004;2(3):188-192.

112. Kadhiravan T, Wig N, Kapil A, Kabra SK, Renuka K, Misra A. Clinical outcomes in typhoid fever: adverse impact of infection with nalidixic acid-resistant Salmonella typhi. *BMC Infect Dis*. 2005;5:37. doi:10.1186/1471-2334-5-37.

113. Walia M, Gaind R, Paul P, Mehta R, Aggarwal P, Kalaivani M. Age-related clinical and microbiological characteristics of enteric fever in India. *Trans R Soc Trop Med Hyg*. 2006;100(10):942-948. doi:10.1016/j.trstmh.2006.02.015.

114. Lakshmi V, Ashok R, Susmita J, Shailaja VV. Changing trends in the antibiograms of Salmonella isolates at a tertiary care hospital in Hyderabad. *Indian J Med Microbiol*. 2006;24(1):45-48. doi:10.4103/0255-0857.19894.

115. Hasan R, Zafar A, Abbas Z, Mahraj V, Malik F, Zaidi A. Antibiotic resistance among Salmonella enterica serovars Typhi and Paratyphi A in Pakistan (2001-2006). *J Infect Dev Ctries*. 2008;2(4):289-294. doi:10.3855/jidc.224.

116. Lewis MD, Serichantalergs O, Pitarangsi C, Chuanak N, Mason CJ, Regmi LR, Pandey P, Laskar R, Shrestha CD, Malla S. Typhoid fever: a massive, single-point source, multidrug-resistant outbreak in Nepal. *Clin Infect Dis*. 2005;40(4):554-561. doi:10.1086/427503.

117. Madhulika U, Harish BN, Parija SC. Current pattern in antimicrobial susceptibility of Salmonella Typhi isolates in Pondicherry. *Indian J Med Res*. 2004;120(2):111-114.

118. Mandal S, Mandal MD, Pal NK. Reduced minimum inhibitory concentration of chloramphenicol for Salmonella enterica serovar typhi. *Indian J Med Sci*. 2004;58(1):16-23.

119. Ray P, Sharma J, Marak RSK, Garg RK. Predictive efficacy of nalidixic acid resistance as a marker of fluoroquinolone resistance in Salmonella enterica var Typhi. *Indian J Med Res*. 2006;124(1):105-108.

120. Malla S, Kansakar P, Serichantalergs O, Rahman M, Basnet S. Epidemiology of typhoid and paratyphoid fever in Kathmandu: two years study and trends of antimicrobial resistance. *J Nepal Med Assoc*. 2005;44(157):18-22.

121. Sharma NP, Peacock SJ, Phumratanaprapin W, Day N, White N, Pukrittayakamee S. A hospital-based study of bloodstream infections in febrile patients in Dhulikhel

Hospital Kathmandu University Teaching Hospital, Nepal. *Southeast Asian J Trop Med Public Health*. 2006;37(2):351-356.

122. Arora D, Singh R, Kaur M, Ahi RS. A changing pattern in antimicrobial susceptibility of Salmonella enterica serotype isolated in North India. *Afr J Microbiol Res*. 2010;4(3):197-203.

123. Joshi S, Adhikary R, Beena HB, Bhavana MV, Bhalwar R. Trends in antibiotic susceptibility of enteric fever isolates from South India, 2002-2013. *Med J Armed Forces India*. 2019;75(1):81-85. doi:10.1016/j.mjafi.2018.08.002.

124. Shirakawa T, Acharya B, Kinoshita S, Kumagai S, Gotoh A, Kawabata M. Decreased susceptibility to fluoroquinolones and gyrA gene mutation in the Salmonella enterica serovar Typhi and Paratyphi A isolated in Katmandu, Nepal, in 2003. *Diagn Microbiol Infect Dis*. 2006;54(4):299-303. doi:10.1016/j.diagmicrobio.2005.10.016.

125. Naheed A, Ram PK, Brooks WA, Hossain MA, Parsons MB, Talukder KA, Mintz E, Luby S, Breiman RF. Burden of typhoid and paratyphoid fever in a densely populated urban community, Dhaka, Bangladesh. *Int J Infect Dis*. 2010;14 Suppl 3:e93-99. doi:10.1016/j.ijid.2009.11.023.

126. Sen B, Dutta S, Sur D, Manna B, Deb AK, Bhattacharya SK, Niyogi SK. Phage typing, biotyping & antimicrobial resistance profile of Salmonella enterica serotype Typhi from Kolkata. *Indian J Med Res*. 2007;125(5):685-688.

127. Holt KE, Dutta S, Manna B, Bhattacharya SK, Bhaduri B, Pickard DJ, Ochiai RL, Ali M, Clemens JD, Dougan G. High-resolution genotyping of the endemic Salmonella Typhi population during a Vi (typhoid) vaccination trial in Kolkata. *PLoS Negl Trop Dis*. 2012;6(1):e1490. doi:10.1371/journal.pntd.0001490.

128. Joshi S, Amarnath SK. Fluoroquinolone resistance in Salmonella typhi and S. paratyphi A in Bangalore, India. *Trans R Soc Trop Med Hyg*. 2007;101(3):308-310. doi:10.1016/j.trstmh.2006.05.009.

129. Maskey AP, Day JN, Phung QT, Thwaites GE, Campbell JI, Zimmerman M, Farrar JJ, Basnyat B. Salmonella enterica serovar Paratyphi A and S. enterica serovar Typhi cause indistinguishable clinical syndromes in Kathmandu, Nepal. *Clin Infect Dis*. 2006;42(9):1247-1253. doi:10.1086/503033.

130. Manchanda V, Bhalla P, Sethi M, Sharma VK. Treatment of enteric fever in children on the basis of current trends of antimicrobial susceptibility of Salmonella enterica serovar typhi and paratyphi A. *Indian J Med Microbiol*. 2006;24(2):101-106. doi:10.4103/0255-0857.25182.

131. Mathura KC, Chaudhary D, Simkhada R, Pradhan M, Shrestha P, Gurubacharya DL. Study of clinical profile and antibiotic sensitivity pattern in culture positive typhoid fever cases. *Kathmandu Univ Med J*. 2005;3(4):376-379.

132. Tamang MD, Oh JY, Seol SY, Kang HY, Lee JC, Lee YC, Cho DT, Kim J. Emergence of multidrug-resistant Salmonella enterica serovar Typhi associated with a class 1 integron carrying the dfrA7 gene cassette in Nepal. *Int J Antimicrob Agents*. 2007;30(4):330-335. doi:10.1016/j.ijantimicag.2007.05.009.

133. Amatya NM, Shrestha B, Lekhak B. Etiological agents of bacteraemia and antibiotic susceptibility pattern in Kathmandu Model Hospital. *J Nepal Med Assoc*. 2007;46(167):112-118.

134. Mirza SH, Khan MA. Low-level quinolone-resistance in multi-drug resistant typhoid. *J Coll Physicians Surg*. 2008;18(1):13-16. doi:01.2008/JCPSP.1316.

135. Kelly DF, Thorson S, Maskey M, Mahat S, Shrestha U, Hamaluba M, Williams E, Dongol S, Werno AM, Portess H, Yadav BK, Adhikari N, Guiver M, Thomas K, Murdoch DR, Pollard AJ. The burden of vaccine-preventable invasive bacterial infections and pneumonia in children admitted to hospital in urban Nepal. *Int J Infect Dis*. 2011;15(1):e17-23. doi:10.1016/j.ijid.2010.05.021.

136. Raveendran R, Wattal C, Sharma A, Oberoi JK, Prasad KJ, Datta S. High level ciprofloxacin resistance in Salmonella enterica isolated from blood. *Indian J Med Microbiol*. 2008;26(1):50-53. doi:10.4103/0255-0857.38858.

137. Sarker AS-E-A, Saha SK, Islam M, Hossain MA. Reduced susceptibility to fluroquinolone and molecular analysis of ciprofloxacine resistance in salmonella typhi in Bangladesh. *Dhaka Univ J Biol Sci*. 2010;19(2):137-144. doi:10.3329/dujbs.v19i2.8955.

138. Vidyalakshmi K, Yashavanth R, Chakrapani M, Shrikala B, Bharathi B, Suchitra U, Dhanashree B, Dominic RMS. Epidemiological shift, seasonal variation and antimicrobial susceptibility patterns among enteric fever pathogens in South India. *Trop Doct*. 2008;38(2):89-91. doi:10.1258/td.2007.070271.

139. Bhattacharya SS, Das U, Choudhury BK. Occurrence & antibiogram of Salmonella Typhi & S. Paratyphi A isolated from Rourkela, Orissa. *Indian J Med Res*. 2011;133:431-433.

140. Rai S, Jain S, Prasad KN, Ghoshal U, Dhole TN. Rationale of azithromycin prescribing practices for enteric fever in India. *Indian J Med Microbiol*. 2012;30(1):30-33. doi:10.4103/0255-0857.93017.

141. Menezes GA, Harish BN, Khan MA, Goessens WHF, Hays JP. Antimicrobial resistance trends in blood culture positive Salmonella Typhi isolates from Pondicherry,

India, 2005-2009. *Clin Microbiol Infect*. 2012;18(3):239-245. doi:10.1111/j.1469-0691.2011.03546.x.

142. Ahmed D, Nahid MA, Sami AB, Halim F, Akter N, Sadique T, Rana MS, Elahi MSB, Rahman MM. Bacterial etiology of bloodstream infections and antimicrobial resistance in Dhaka, Bangladesh, 2005-2014. *Antimicrob Resist Infect Control*. 2017;6:2. doi:10.1186/s13756-016-0162-z.

143. Sharma P, Dahiya S, Manral N, Kumari B, Kumar S, Pandey S, Sood S, Das BK, Kapil A. Changing trends of culture-positive typhoid fever and antimicrobial susceptibility in a tertiary care North Indian Hospital over the last decade. *Indian J Med Microbiol*. 2018;36(1):70-76. doi:10.4103/ijmm.IJMM_17_412.

144. Abbasi S, Imtiaz A, Usman J, Kaleem F, Hassan A. Evaluation of the current trend of nalidixic acid susceptibility in typhoidal Salmonellae; a marker of therapeutic failure for the fluoroquinolones. *Iran J Microbiol*. 2011;3(2):80-83.

145. Prajapati B, Rai GK, Rai SK, Upreti HC, Thapa M, Singh G, Shrestha RM. Prevalence of Salmonella typhi and Paratyphi infection in children: a hospital based study. *Nepal Med Coll J*. 2008;10(4):238-241.

146. Gopal M, Elumalai S, Arumugam S, Durairajpandian V, Kannan MA, Selvam E, Seetharaman S. GyrA ser83 and ParC trp106 Mutations in Salmonella enterica Serovar Typhi Isolated from Typhoid Fever Patients in Tertiary Care Hospital. *J Clin Diagn Res*. 2016;10(7):DC14-18. doi:10.7860/JCDR/2016/17677.8153.

147. Elumalai S, Muthu G, Selvam REM, Ramesh S. Detection of TEM-, SHV- and CTX-M-type β-lactamase production among clinical isolates of Salmonella species. *J Med Microbiol*. 2014;63(Pt 7):962-967. doi:10.1099/jmm.0.068486-0.

148. Iyer RN, Jangam RR, Jacinth A, Venkatalakshmi A, Nahdi FB. Prevalence and trends in the antimicrobial susceptibility pattern of Salmonella enterica serovars Typhi and Paratyphi A among children in a pediatric tertiary care hospital in South India over a period of ten years: a retrospective study. *Eur J Clin Microbiol Infect Dis*. 2017;36(12):2399-2404. doi:10.1007/s10096-017-3073-x.

149. Acharya D, Bhatta DR, Malla S, Dumre SP, Adhikari N, Kandel BP. Salmonella enterica serovar Paratyphi A: an emerging cause of febrile illness in Nepal. *Nepal Med Coll J*. 2011;13(2):69-73.

150. Ali SQ, Zehra A, Naqvi BS, Shah S, Bushra R. Resistance pattern of ciprofloxacin against different pathogens. *Oman Med J*. 2010;25(4):294-298. doi:10.5001/omj.2010.85.

151. Kumar Y, Sharma A, Mani KR. Antibiogram profile of Salmonella enterica serovar Typhi in India - a two year study. *Trop Life Sci Res*. 2013;24(1):45-54.

152. Pokharel P, Rai SK, Karki G, Katuwal A, Vitrakoti R, Shrestha SK. Study of enteric fever and antibiogram of Salmonella isolates at a teaching hospital in Kathmandu Valley. *Nepal Med Coll J*. 2009;11(3):176-178.

153. Gupta V, Singla N, Bansal N, Kaistha N, Chander J. Trends in the antibiotic resistance patterns of enteric Fever isolates - a three year report from a tertiary care centre. *Malays J Med Sci*. 2013;20(4):71-75.

154. Bandyopadhyay R, Balaji V, Yadav B, Jasmine S, Sathyendra S, Rupali P. Effectiveness of treatment regimens for Typhoid fever in the nalidixic acid-resistant S. typhi (NARST) era in South India. *Trop Doct*. 2018;48(3):182-188. doi:10.1177/0049475518758884.

155. Britto CD, Dyson ZA, Duchene S, Carter MJ, Gurung M, Kelly DF, Murdoch DR, Ansari I, Thorson S, Shrestha S, Adhikari N, Dougan G, Holt KE, Pollard AJ. Laboratory and molecular surveillance of paediatric typhoidal Salmonella in Nepal: Antimicrobial resistance and implications for vaccine policy. *PLoS Negl Trop Dis*. 2018;12(4):e0006408. doi:10.1371/journal.pntd.0006408.

156. Alam MS, Pillai PK, Kapur P, Pillai KK. Resistant patterns of bacteria isolated from bloodstream infections at a university hospital in Delhi. *J Pharm Bioallied Sci*. 2011;3(4):525-530. doi:10.4103/0975-7406.90106.

157. Acharya D, Trakulsomboon S, Madhup SK, Korbsrisate S. Antibiotic susceptibility pattern and the indicator of decreased ciprofloxacin susceptibility of Salmonella enterica serovar Typhi isolated from Dhulikhel Hospital, Nepal. *Jpn J Infect Dis*. 2012;65(3):264-267. doi:10.7883/yoken.65.264.

158. Chandrasiri P, Elwitigala J, Nanayakkara G, Chandrasiri S. A multi centre laboratory study of Gram negative bacterial blood stream infections in Sri Lanka. *Ceylon Med J*. 2013;58(2):56-61. doi:10.4038/cmj.v58i2.5680.

159. Singla N, Bansal N, Gupta V, Chander J. Outbreak of Salmonella Typhi enteric fever in sub-urban area of North India: a public health perspective. *Asian Pac J Trop Med*. 2013;6(2):167-168. doi:10.1016/S1995-7645(13)60017-6.

160. Choudhary A, Gopalakrishnan R, Nambi PS, Ramasubramanian V, Ghafur KA, Thirunarayan MA. Antimicrobial susceptibility of Salmonella enterica serovars in a tertiary care hospital in southern India. *Indian J Med Res*. 2013;137(4):800-802.

161. Qamar FN, Azmatullah A, Kazi AM, Khan E, Zaidi AKM. A three-year review of antimicrobial resistance of Salmonella enterica serovars Typhi and Paratyphi A in Pakistan. *J Infect Dev Ctries*. 2014;8(8):981-986. doi:10.3855/jidc.3817.

162. Shetty AK, Shetty IN, Furtado ZV, Antony B, Boloor R. Antibiogram of salmonella isolates from blood with an emphasis on nalidixic Acid and chloramphenicol susceptibility in a tertiary care hospital in coastal karnataka: a prospective study. *J Lab Physicians*. 2012;4(2):74-77. doi:10.4103/0974-2727.105585.

163. Dutta S, Das S, Mitra U, Jain P, Roy I, Ganguly SS, Ray U, Dutta P, Paul DK. Antimicrobial resistance, virulence profiles and molecular subtypes of Salmonella enterica serovars Typhi and Paratyphi A blood isolates from Kolkata, India during 2009-2013. *PloS One*. 2014;9(8):e101347. doi:10.1371/journal.pone.0101347.

164. Afzal A, Sarwar Y, Ali A, Maqbool A, Salman M, Habeeb MA, Haque A. Molecular evaluation of drug resistance in clinical isolates of Salmonella enterica serovar Typhi from Pakistan. *J Infect Dev Ctries*. 2013;7(12):929-940. doi:10.3855/jidc.3154.

165. Geetha VK, Yugendran T, Srinivasan R, Harish BN. Plasmid-mediated quinolone resistance in typhoidal Salmonellae: a preliminary report from South India. *Indian J Med Microbiol*. 2014;32(1):31-34. doi:10.4103/0255-0857.124292.

166. Raza S, Tamrakar R, Bhatt CP, Joshi SK. Antimicrobial susceptibility patterns of Salmonella Typhi and Salmonella Paratyphi A in a tertiary care hospital. *J Nepal Health Res Counc*. 2012;10(22):214-217.

167. Jain S, Das Chugh T. Antimicrobial resistance among blood culture isolates of Salmonella enterica in New Delhi. *J Infect Dev Ctries*. 2013;7(11):788-795. doi:10.3855/jidc.3030.

168. Yadav VC, Kiran VR, Sharma R. Enteric fever in Bastar tribal region-prevalence and sensitivity patterns. *J Evol Med Dent Sci*. 2005;5. doi:DOI: 10.14260/jemds/2016/813.

169. Vala S, Shah U, Ahmad SA, Scolnik D, Glatstein M. Resistance patterns of typhoid fever in children: a longitudinal community-based study. *Am J Ther*. 2016;23(5):e1151-1154. doi:10.1097/MJT.0000000000000094.

170. Chand HJ, Rijal KR, Neupane B, Sharma VK, Jha B. Re-emergence of susceptibility to conventional first line drugs in Salmonella isolates from enteric fever patients in Nepal. *J Infect Dev Ctries*. 2014;8(11):1483-1487. doi:10.3855/jidc.4228.

171. Ikram S, Hussain S, Aslam A, Khan MD, Ahmed I. Evaluation of the current trends in the antimicrobial susceptibility patterns of typhoid salmonellae. *Pak J Med Health Sci*. 2016;10(1):307-312.

172. Sania KM, Shyamasakhi PD, Pramodini KD, Sulochana KD. Evaluation of minimum inhibitory concentration of chloramphenicol for salmonella spp. Isolated from enteric fever cases in a tertiary hospital in Imphal. *Int J Pharm Sci Res*. 2016;7(9):3815-3819.

173. Makkar A, Gupta S, Khan ID, Gupta RM, Rajmohan KS, Chopra H, Gupta M, Bansal S, Poonia B, Malik M, Panda PS. Epidemiological profile and antimicrobial resistance pattern of enteric fever in a tertiary care hospital of north India - a seven year ambispective study. *Acta Medica*. 2018;61(4):125-130. doi:10.14712/18059694.2018.130.

174. Roka G, Pandaya S, Ferdous MR, Pandey M, Pokhrel NR, Shrestha R, Alam TT, Billah MM, Ahamed SK. Susceptibility to patterns of ciprofloxacin among nalidixic acid-resistant Salmonella isolates collected in Banepa, Nepal from enteric fever patients. *J Biol Res*. 2016;89(2).

175. Garg A, Verma S, Kanga A, Singh D, Singh B. Antimicrobial resistance pattern and in vivo activity of azithromycin in Salmonella isolates. *Indian J Med Microbiol*. 2013;31(3):287.

176. Shrestha KL, Pant ND, Bhandari R, Khatri S, Shrestha B, Lekhak B. Re-emergence of the susceptibility of the Salmonella spp. isolated from blood samples to conventional first line antibiotics. *Antimicrob Resist Infect Control*. 2016;5:22. doi:10.1186/s13756-016-0121-8.

177. Srirangaraj S, Kali A, Charles MVP. A study of antibiogram of Salmonella enterica serovar Typhi isolates from Pondicherry, India. *Australas Med J*. 2014;7(4):185-190. doi:10.4066/AMJ.2014.2010.

178. Ali A, Ali HA, Shah FH, Zahid A, Aslam H, Javed B. Pattern of antimicrobial drug resistance of Salmonella Typhi and Paratyphi A in a Teaching Hospital in Islamabad. *J Pak Med Assoc*. 2017;67(3):375-379.

179. Bakthavatchalam YD, Kumar DT, Tayubi IA, Shankar BA, Babu P, Munusamy E, Thukkaram B, Ravi R, Doss CGP, Veeraraghavan B. In vitro efficacy and in silico analysis of cefixime-ofloxacin combination for Salmonella Typhi from bloodstream infection. *J Appl Microbiol*. 2017;123(3):615-624. doi:10.1111/jam.13522.

180. Petersiel N, Shresta S, Tamrakar R, Koju R, Madhup S, Shresta A, Bedi T, Zmora N, Paran Y, Schwartz E, Neuberger A. The epidemiology of typhoid fever in the Dhulikhel area, Nepal: A prospective cohort study. *PloS One*. 2018;13(9):e0204479. doi:10.1371/journal.pone.0204479.

181. Qamar FN, Yousafzai MT, Sultana S, Baig A, Shakoor S, Hirani F, Wassay A, Khushboo S, Mehmood J, Freeman A, Date K, Garrett D. A retrospective study of laboratory-based enteric fever surveillance, Pakistan, 2012-2014. *J Infect Dis*. 2018;218(suppl_4):S201-S205. doi:10.1093/infdis/jiy205.

182. Sharvani R, Hemavathi null, Dayanand DK, Shenoy P, Sarmah P. Antibiogram of Salmonella isolates: time to consider antibiotic salvage. *J Clin Diagn Res*. 2016;10(5):DC06-08. doi:10.7860/JCDR/2016/18102.7753.

183. Ejaz SK, Poddar CK, Singh MN, Hasan AR, Prasad J, Kumar R, Bhowmick N, Sinha RN. Antimicrobial susceptibility of salmonella enterica serovars in a tertiary care hospital in Koshi region (Northern Bihar), India. *J Evol Med Dent Sci*. 2017;6(22):1808-1811.

184. Misra R, Thakare R, Amrin N, Prasad KN, Chopra S, Dhole TN. Antimicrobial susceptibility pattern and sequence analysis of DNA gyrase and DNA topoisomerase IV in Salmonella enterica serovars Typhi and Paratyphi A isolates with decreased susceptibility to ciprofloxacin. *Trans R Soc Trop Med Hyg*. 2016;110(8):472-479. doi:10.1093/trstmh/trw051.

185. Ali MK, Sultana S. Antimicrobial sensitivity patterns of salmonella typhi in children. *Bangladesh J Med Sci*. 2016;15(3):416-418. doi:10.3329/bjms.v15i3.30198.

186. Erum S, Fasih F, Fatima A. Frequency of salmonella typhi among bacteremic isolates and their susceptibility pattern against azithromycin. *Rawal Med J*. 2019;44(1):4-6.

187. Zehra NM, Irfan F, Mirza IA, Imtiaz A, Nadeem S, Hameed F. Current trends of antimicrobial susceptibility of typhoidal Salmonellae isolated at tertiary care hospital. *J Coll Physicians Surg*. 2017;27(11):690-692. doi:2744.

188. Purighalla S, Esakimuthu S, Reddy M, Seth T, Patil SD, Varghese GK, Dasarathy R, Richard VS, Sambandamurthy VK. Investigation into a community outbreak of Salmonella Typhi in Bengaluru, India. *Indian J Med Res*. 2017;146(Supplement):S15-S22. doi:10.4103/ijmr.IJMR_1201_16.

189. Bhetwal A, Maharjan A, Khanal PR, Parajuli NP. Enteric fever caused by Salmonella enterica serovars with reduced susceptibility of fluoroquinolones at a community based teaching hospital of Nepal. *Int J Microbiol*. 2017;2017:2869458. doi:10.1155/2017/2869458.

190. Khanal PR, Satyal D, Bhetwal A, Maharjan A, Shakya S, Tandukar S, Parajuli NP. Renaissance of conventional first-line antibiotics in Salmonella enterica clinical isolates: assessment of MICs for therapeutic antimicrobials in enteric fever cases from Nepal. *BioMed Res Int*. 2017;2017:2868143. doi:10.1155/2017/2868143.

191. Imran M, Dost S, Saleem M. Pattern of antibiotic resistance among patients with enteric fever. *Indo Am J Pharm Sci*. 2018;5(8):7797-7801.

192. Sucindar M, Kumaran SS. Profile of culture positive enteric fever in children admitted in a tertiary care hospital. *J Evol Med Dent Sci*. 2017;6(88):6112-6117.

193. Shaikh AA, Shaikh A, Tahir A. Antimicrobial resistance trends of typhoidal salmonellae in southern Pakistan. *Rawal Med J*. 2019;44(1):7-10.

194. Laghari GS, Hussain Z, Hussain SZM, Kumar H, Uddin SMM, Haq A. Antimicrobial Susceptibility Patterns of Salmonella Species in Southern Pakistan. *Cureus*. 2019;11(4).

195. Hamze M, Dabboussi F, Izard D. [Enterobacterial susceptibility to antibiotics in northern Lebanon (1998-2001)]. *Sante*. 2003;13(2):107-112.

196. Dimitrov T, Dashti AA, Albaksami O, Jadaon MM. Detection of mutations in the gyrA gene in fluoroquinolone resistance Salmonella enterica serotypes typhi and paratyphi A isolated from the Infectious Diseases Hospital, Kuwait. *J Clin Pathol*. 2010;63(1):83-87. doi:10.1136/jcp.2009.070664.

197. Abro AH, Al Deesi ZO, Abdou AMS. Salmonella typhi: Antibiotic sensitivity pattern in Dubai, United Arab Emirates. 2011.

198. Papaevangelou V, Syriopoulou V, Charissiadou A, Pangalis A, Mostrou G, Theodoridou M. Salmonella bacteraemia in a tertiary children's hospital. *Scand J Infect Dis*. 2004;36(8):547-551. doi:10.1080/00365540410016744.
