# Supplementary material for: A Systematic Review on Antimicrobial Resistance among Salmonella Typhi Worldwide
Source: Am J Trop Med Hyg. 2020 Sep 28;103(6):2518–27. doi: 10.4269/ajtmh.20-0258 (PMC7695120; doi:10.4269/ajtmh.20-0258)
Supplement: Supplementary file 2 [file tpmd200258.SD2.docx]

**Table S3** Non-typhi AMR

**Table S4** Data for figures

*Note*: Table S3 and S4 will be available online in final publication.
